# Vancomycin-resistant *Staphylococcus aureus* (VRSA) can overcome the cost of antibiotic resistance and may threaten vancomycin’s clinical durability

**DOI:** 10.1371/journal.ppat.1012422

**Published:** 2024-08-29

**Authors:** Samuel E. Blechman, Erik S. Wright

**Affiliations:** 1 Department of Biomedical Informatics, University of Pittsburgh, Pittsburgh, Pennsylvania, United States of America; 2 Center for Evolutionary Biology and Medicine, Pittsburgh, Pennsylvania, United States of America; University of Mississippi Medical Center, UNITED STATES OF AMERICA

## Abstract

Vancomycin has proven remarkably durable to resistance evolution by *Staphylococcus aureus* despite widespread treatment with vancomycin in the clinic. Only 16 cases of vancomycin-resistant *S*. *aureus* (VRSA) have been documented in the United States. It is thought that the failure of VRSA to spread is partly due to the fitness cost imposed by the *vanA* operon, which is the only known means of high-level resistance. Here, we show that the fitness cost of *vanA*-mediated resistance can be overcome through laboratory evolution of VRSA in the presence of vancomycin. Adaptation to vancomycin imposed a tradeoff such that fitness in the presence of vancomycin increased, while fitness in its absence decreased in evolved lineages. Comparing the genomes of vancomycin-exposed and vancomycin-unexposed lineages pinpointed the D-alanine:D-alanine ligase gene (*ddl*) as the target of loss-of-function mutations, which were associated with the observed fitness tradeoff. Vancomycin-exposed lineages exhibited vancomycin dependence and abnormal colony morphology in the absence of drug, which were associated with mutations in *ddl*. However, further evolution of vancomycin-exposed lineages in the absence of vancomycin enabled some evolved lineages to escape this fitness tradeoff. Many vancomycin-exposed lineages maintained resistance in the absence of vancomycin, unlike their ancestral VRSA strains. These results indicate that VRSA might be able to compensate for the fitness deficit associated with *vanA*-mediated resistance, which may pose a threat to the prolonged durability of vancomycin in the clinic. Our results also suggest vancomycin treatment should be immediately discontinued in patients after VRSA is identified to mitigate potential adaptations.

## Author summary

The clinical utility of antibiotics is threatened by the potential evolution of resistance in the pathogen being targeted. Thus, we seek to understand why some antibiotics effectively evade the evolution of resistance. Vancomycin has remained one of the few effective antibiotics in the treatment of methicillin-resistant *Staphylococcus aureus* (MRSA), with few cases of vancomycin-resistant *S*. *aureus* (VRSA) being reported. However, the basis of vancomycin’s durability and the ability of VRSA to further adapt to vancomycin in the laboratory has yet to be investigated. Herein, we describe the evolutionary trajectories of several laboratory-propagated VRSA strains. We show that VRSA can overcome the fitness cost imposed by vancomycin resistance, a result which threatens the long-term use of vancomycin in the clinic.

## Introduction

Few antibiotics have proven durable to the evolution of resistance in the face of their widespread use to treat pathogens. Famous examples include continued penicillin susceptibility in Group A *Streptococcus* [1] and vancomycin susceptibility in *Staphylococcus aureus* [2,3], where resistance has failed to proliferate despite extensive selection pressure. Such cases of antibiotic durability may hold the key to developing antibiotics that evade the evolution of resistance [4–8]. It is believed that widespread resistance fails to emerge because of the fitness cost imposed by resistance, the scarcity of viable resistance mechanisms, or the incompatibility of resistance conferring mobile genetic elements across pathogens [5,9]. In the case of *S*. *aureus*, the basis of vancomycin durability remains unclear and, therefore, it is unknown whether the evolution of vancomycin resistance will pose a major long-term threat in the clinic.

The continued success of vancomycin for the treatment of methicillin-resistant *S*. *aureus* (MRSA) is particularly intriguing because vancomycin has remained the first-line therapy for 40 years [10]. MRSA is responsible for about 70,000 infections per year in the United States alone [11], yet only 16 cases of vancomycin-resistant *S*. *aureus* (VRSA) have been detected in the US [12]. Several cases of VRSA were reported across the world in recent years, including Brazil, India, Iran, Pakistan, and Portugal [3]. Thus far, VRSA has failed to spread between patients, in sharp contrast to vancomycin-resistant *Enterococcus* (VRE) [13]. MRSA acquired resistance to multiple drugs prior to the widespread usage of vancomycin, and also evolved *de novo* resistance to the more recent antibiotics linezolid [14,15] and daptomycin [16,17]. While *de novo* mutations imparting intermediate-level resistance to vancomycin (minimum inhibitory concentrations [MICs] of 4–8 μg/ml) are observed at low rates in the clinic [18,19], all documented cases of VRSA have been plasmid-mediated through horizontal gene transfer of the *vanA* operon from VRE during coinfection [20–29].

During normal peptidoglycan synthesis, pentaglycyl lipid II precursors enter the extracellular matrix by the action of flippase and participate in transglycosylase and transpeptidase reactions by penicillin-binding proteins (PBPs) to form mature peptidoglycan. Vancomycin leads to cell death by binding the D-alanyl-D-alanine C-termini of pentaglycyl lipid II, inhibiting the transglycosylase action of PBP enzymes [30,31]. The plasmid-encoded *vanA* operon imparts resistance by modifying the C-termini of peptidoglycan precursors to D-alanyl-D-lactate and consists of the genes *vanRSHAXYZ*. VanRS is a two-component regulatory system that activates transcription of the remaining genes when vancomycin enters the periplasmic space. The remaining genes encode enzymes that synthesize D-lactate (*vanH*) and D-alanyl-D-lactate (*vanA*), hydrolyze free D-alanyl-D-alanine (*vanX*), and hydrolyze the D-alanyl-D-alanine termini of UDP-MurNAc-pentapeptide (*vanY*) [32]. The modified pentadepsipeptide precursors have a ~1000-fold lower binding affinity for vancomycin [33]. Unlike VRE, VRSA has only emerged in patients with severe underlying conditions [3,34], which might provide an environment that is permissive to pathogens with lower fitness. Clinical VRSA isolates have a lower growth rate and longer lag time when exposed to vancomycin [35–38]. Several clinical VRSA and VRE strains isolated from patients treated with vancomycin for extended periods exhibit reduced growth in the absence of vancomycin (i.e., vancomycin dependence) and have loss-of-function mutations in the chromosomal gene encoding D-alanine:D-alanine ligase (*ddl*) [37,39–43]. The function of Ddl is opposed by VanX, suggesting that interference with Ddl could be the basis for the fitness cost of *vanA*-mediated resistance.

The fitness cost of antibiotic resistance can often be alleviated by reversion to susceptibility or the acquisition of compensatory mutations that restore fitness without loss of resistance [4,44–47]. In the absence of antibiotic, fitness-compensated resistant mutants may arise more quickly than antibiotic-susceptible revertants due to a greater number of compensatory mutations than reversion mutations [46]. Additionally, compensatory mutations may create an "adaptive valley" between resistant and susceptible genotypes, thus contributing to the irreversibility of resistance [4,44]. In the case of vancomycin-intermediate *S. aureus* (VISA), even if the resistant phenotype is lost, prior development of vancomycin resistance can potentiate future resistance evolution [48]. The fitness cost of antibiotic resistance in *S*. *aureus* has been described for many antibiotics, as well as the compensatory mutations that occur in response [49]. However, whether VRSA can compensate for the fitness cost of vancomycin resistance remains to be explored. The potential for VRSA to overcome this fitness deficit through the acquisition of compensatory mutations poses a major threat to public health due to the high mortality rate of MRSA and the limited number of effective antibiotics.

We set out to determine whether VRSA can adapt to growth in vancomycin and compensate for the fitness cost imposed by *vanA*-mediated resistance. To this end, we propagated multiple clinical VRSA isolates in the presence (VAN-exposed) and absence (VAN-unexposed) of vancomycin and tracked their fitness during evolution. Using high-throughput sequencing we were able to pinpoint mutations that compensate for the fitness cost associated with the *vanA* operon. Furthermore, we sought to determine whether the observed mutations precluded VRSA from reverting to susceptibility in the absence of vancomycin. Our results shed light on why some antibiotics remain durable despite continual selection for resistance and provide insights into why many pathogens remain resistant to antibiotics long after their use is discontinued in the clinic [4,44–47,50–54].

## Results

**Fig 1:**
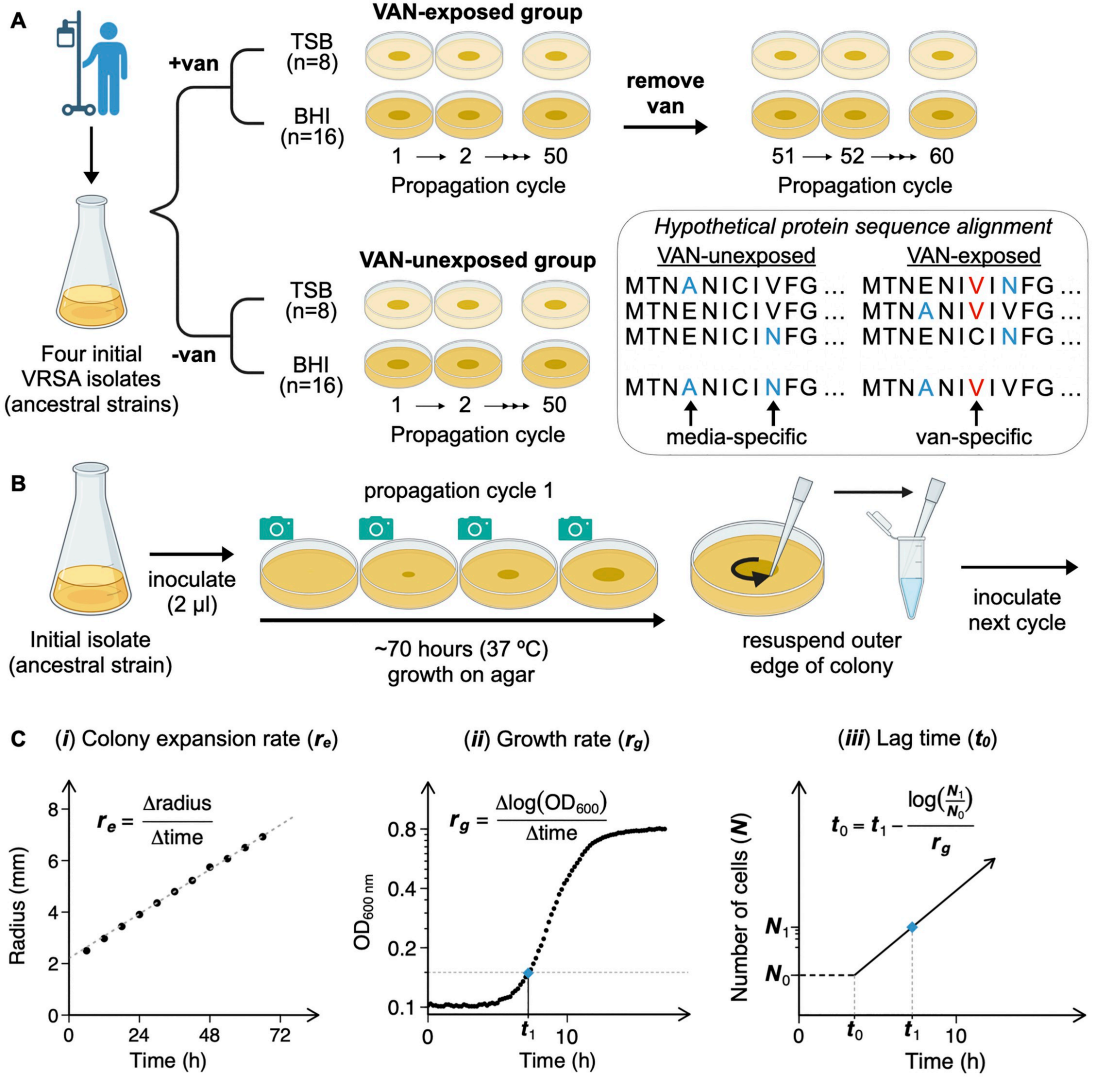
Highly parallel laboratory evolution of clinical VRSA isolates. (**A**) Four VRSA strains were propagated in parallel on BHI and TSB agar media with vancomycin (32 μg/ml) and without. This was repeated for 50 propagation cycles at 37°C for ~70 h of growth per cycle. VAN-exposed lineages were propagated for a further 10 cycles [51–60] on vancomycin-free agar. DNA sequencing of ancestral strains and cycle 50 and 60 evolved lineages was performed to pinpoint mutations specific to vancomycin, rather than the growth media. (**B**) Following growth, the outer edge of each colony was picked using a pipette tip and resuspended in 60% glycerol. Two μl of the resuspension solution was used to inoculate the next propagation cycle. (**C**) Three growth measures used as proxies for fitness (see Materials and Methods): (***i***) Colony expansion rate (***r***_***e***_): colonies were scanned every 6 h during growth and the radius of each colony was measured from the images. The ***r***_***e***_ is the slope of a linear model fit to the points (see Materials and Methods). (***ii***) Optical density growth curves were captured and growth rate (***r***_***g***_) was calculated from the slope of the log of OD_600_. (***iii***) Lag time (***t***_***0***_) was calculated using the delay time (***t***_***1***_—see panel [ii] and Materials and Methods) and growth rate (***r***_***g***_), as well as estimated population size at inoculation (***N***_***0***_) and at delay time (***N***_***1***_). Note logarithmic scaled y-axis in (ii) and (iii). Fig 1A and 1B were made using *Biorender*.*com*.

We selected four clinical VRSA isolates (VRSA-3a, -4, -6, and -10; strains HIP13170, HIP14300, AIS2006032, and AIS 1000505, respectively) for propagation based on their susceptibility to multiple antibiotics. We verified that all four strains are resistant to vancomycin and oxacillin and susceptible to linezolid and sulfamethoxazole-trimethoprim (S8 Fig). All four strains have the same multi-locus sequence type profile (ST 5) and harbor the *vanA* operon on a plasmid. VRSA-4, -6, and -10 are USA100 isolates with SCC*mec* subtype II, while VRSA-3a is a USA800/Pediatric isolate with SCC*mec* subtype IV. VRSA-3a was co-isolated with VRSA-3b. VRSA-3a was chosen for experimentation and will be referred to as VRSA-3 hereafter [24]. VRSA strains were grown on solid medium for 50 propagation cycles of ~70 h of growth (Fig 1A). Each propagation cycle corresponded to 10 or more generations, resulting in a total of at least 500 generations. Lineages propagated on agar containing vancomycin were propagated for an additional 10 cycles on vancomycin-free agar. Solid medium was chosen because it allows colonies to be visually monitored and maintain exponential growth phase for a longer duration than liquid medium. Colonies grew from the center of agar plates that were laid on flatbed scanners in a 37°C incubator and scanned every 6 hours, allowing the expansion rate of each independent lineage to be measured (Fig 1B and 1C). Each strain was independently propagated in 8 or 16 parallel lineages in both the presence (32 μg/ml) and absence of vancomycin on two media (Fig 1A and 1B). Brain heart infusion (BHI) and tryptic soy broth without dextrose (TSB) were chosen to reflect alternative growth environments.

### Most clinical VRSA isolates are poorly adapted to growth in the presence of vancomycin

**Fig 2:**
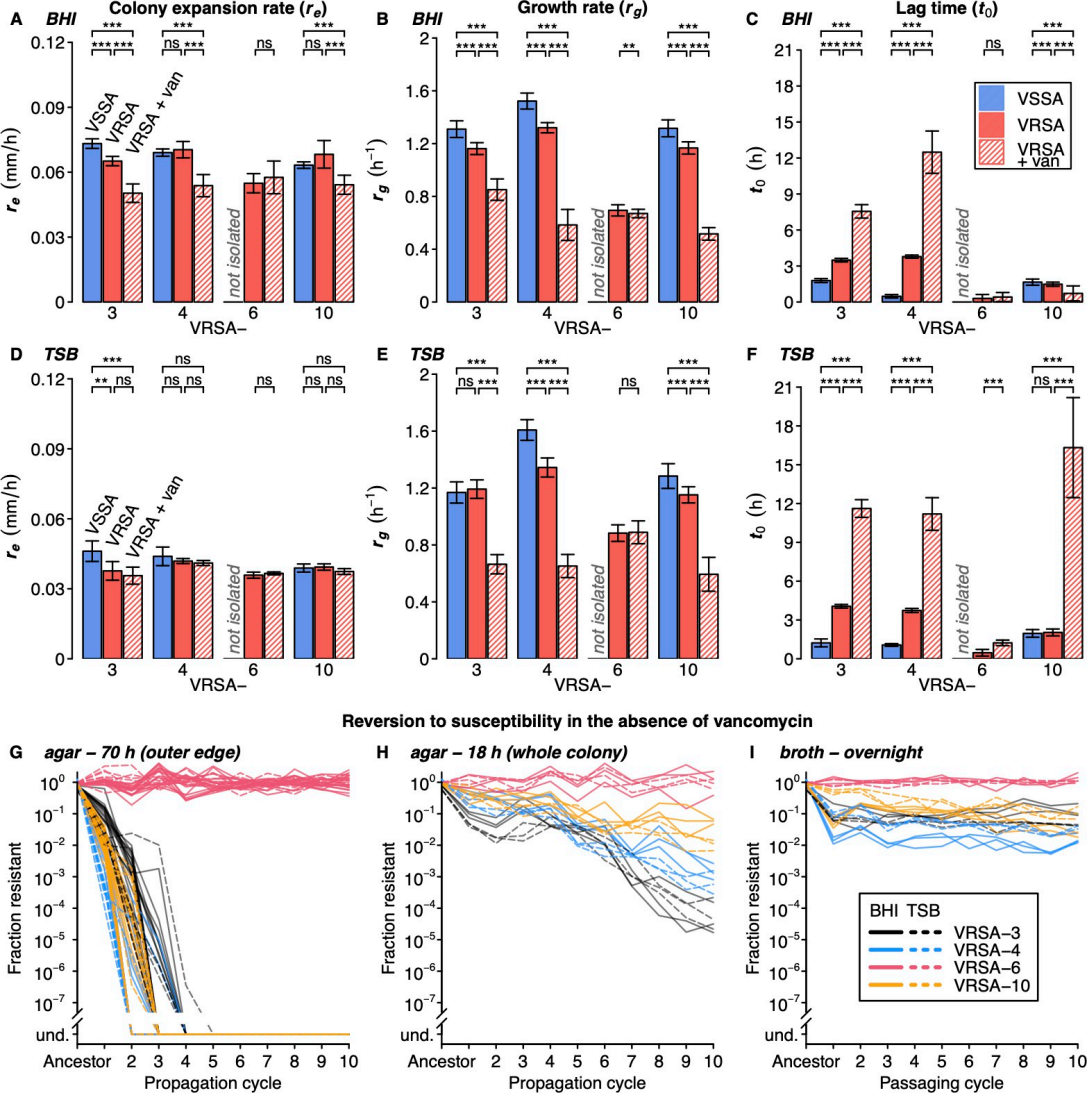
The initial fitness cost and stability of vancomycin resistance differed by medium and strain. (**A-F**) Growth measurements of ancestral VRSA strains and their respective VSSA strain (where applicable) in BHI (**A-C**) and TSB (**D-F**) on solid media (n = 16 on BHI and n = 8 on TSB) and in liquid media (n = 96). (**A** and **D**) VRSA-4 and -10 had a similar colony expansion rate (***r*_*e*_**) to their respective VSSA strains, while VRSA-3 grew slower than VSSA-3. VRSA-3, -4, and -10 had a slower ***r*_*e*_** in the presence of vancomycin on BHI, but not TSB agar medium. VSSA-6 was not isolated and the ***r*_*e*_** of VRSA-6 did not differ in the presence of vancomycin. (**B** and **E**) VRSA-3, -4, and -10 had a slower growth rate (***r*_*g*_**) than their respective VSSA strains, except for VRSA-3 in TSB liquid medium. All VRSA strains had a slower ***r*_*g*_** in the presence of vancomycin, except for VRSA-6 in TSB liquid medium. (**C** and **F**) VRSA-3 and -4 had a longer lag time (***t*_*0*_**) than their respective VSSA strains. All VRSA strains had a longer ***t*_*0*_** in the presence of vancomycin, except for VRSA-10 in BHI liquid medium. Error bars represent standard deviation. Differences between groups were compared by two-sided Wilcoxon rank-sum test and asterisks denote statistical significance after Holm-Bonferroni multiple testing correction (*** = p-adj. < 0.001, ** = p-adj. < 0.01, * = p-adj. < 0.05, ns = p-adj. ≥ 0.05). (**G**) Three of four VRSA strains completely reverted to susceptibility during propagation on vancomycin-free agar medium (und. = no resistant cells detected). Reversion occurred in BHI (n = 16) and TSB (n = 8) media across all replicates. VRSA-6 maintained resistance in the absence of vancomycin through 10 cycles in all cases. (**H-I**) VRSA-3, -4, and -10 underwent partial reversion to susceptibility when whole colonies were propagated on vancomycin-free agar (**H**) and when passaged in vancomycin-free liquid medium (**I**) (n = 3 in BHI and n = 3 in TSB).

We first sought to determine whether *vanA*-mediated resistance imposed a fitness cost in the presence of vancomycin. To this end, we estimated the baseline fitness of ancestral strains through multiple proxies: colony expansion rate (***r***_***e***_) on solid (agar) medium, as well as growth rate (***r***_***g***_) and lag time (***t***_***0***_) in liquid (broth) medium (Fig 1C). The effect of vancomycin exposure on fitness proxies differed by strain, growth medium, and mode of growth (i.e., solid or liquid). VRSA-3, -4, and -10 exhibited a sharp reduction in colony expansion rate and growth rate in the presence of vancomycin, with the exception of colony expansion rate on TSB agar (Fig 2A, 2B and 2D–2E). The colony expansion rate and growth rate of VRSA-6 did not substantially differ in the presence of vancomycin (Fig 2A and 2B). The observed growth rate reduction in vancomycin for VRSA-3 and VRSA-10 is consistent with previously published data on these strains [35–37]. With few exceptions, all VRSA strains tested showed an increased lag time in the presence of vancomycin (Fig 2C and 2F), corroborating previous studies [35,36,38].

Due to the instability of resistance, we were able to isolate vancomycin-susceptible *S*. *aureus* (VSSA) isolates of VRSA-3, -4, and -10 after one propagation cycle, but not VRSA-6. VSSA strains generally showed greater fitness than their resistant counterparts (Fig 2A–2F). These results indicate that most VRSA isolates are poorly adapted to growth in a physiologically-relevant vancomycin concentration and estimate the fitness cost of *vanA*-mediated resistance [55]. However, we did not investigate the role of individual plasmid components in the fitness cost observed in VRSA.

### Most VRSA quickly revert to VSSA in the absence of vancomycin pressure

All lineages of VRSA-3, -4, and -10 grown in the absence of vancomycin reverted to susceptibility within 2 to 5 propagation cycles on solid media (Fig 2G). We use the phrase “reversion to (vancomycin) susceptibility” hereafter in reference to VRSA lineages that do not grow on agar medium (BHI or TSB) with 32 μg/ml vancomycin, rather than in the strict sense of an antibiotic susceptibility test result. Reversion to susceptibility occurred simultaneously with loss of *vanA* (S1 Fig). Whole genome sequencing revealed the *vanA* operon and surrounding region (i.e., the entire contig) were absent at propagation cycle 50 in nearly all VAN-unexposed lineages (S2A Fig), suggesting reversion to susceptibility occurs due to segregational loss of the plasmid on which the *vanA* operon resides. In contrast, VRSA-6 maintained resistance through 50 propagation cycles in all but one lineage (of 24) that underwent reversion to susceptibility between propagation cycles 17 and 23. This lineage maintained the *vanA* operon but had a nonsense mutation in *vanX* (S2A Fig). These results indicate heterogeneity in the stability of resistance among VRSA strains, corroborating previous studies [35,36].

We passaged the same four ancestral VRSA strains in vancomycin-free liquid media for 10 cycles of overnight growth. For consistency, we matched the liquid inoculum and final population sizes to those on solid agar medium (~10^7^ and ~10^10^ colony forming units [CFUs], respectively). Interestingly, VRSA-3, -4, and -10 underwent partial reversion to susceptibility during the first few passaging cycles in liquid media but retained a stable subpopulation (~1–10%) of resistant cells in the absence of vancomycin (Fig 2I). We hypothesized that this difference across media was due to features of the propagation techniques. That is, in broth a fraction of the total liquid volume is transferred, while, on agar only the outer edge of each grown colony is transferred to the next cycle. The colony perimeter presumably contains cells only from the most recent generations and is likely dominated by fitness-advantaged susceptible cells. Additionally, population size in broth medium exponentially increases and reaches saturation after ~10 generations, whereas population size on agar increases approximately quadratically for ~70 hours. Thus, growth on agar medium likely allows for more than 10 generations per cycle. To more closely match the agar and broth conditions, we propagated colonies on agar by resuspending the whole colony after 18 hours of growth and transferring a fraction of the whole colony to the next cycle. Much like broth propagation, this resulted in only partial reversion to susceptibility for VRSA-3, -4, and -10 after 10 cycles (Fig 2H). To our knowledge, this is the first demonstration of a difference in reversion to susceptibility between solid and liquid medium.

### VAN-exposed lineages exhibit a fitness tradeoff associated with vancomycin

**Fig 3:**
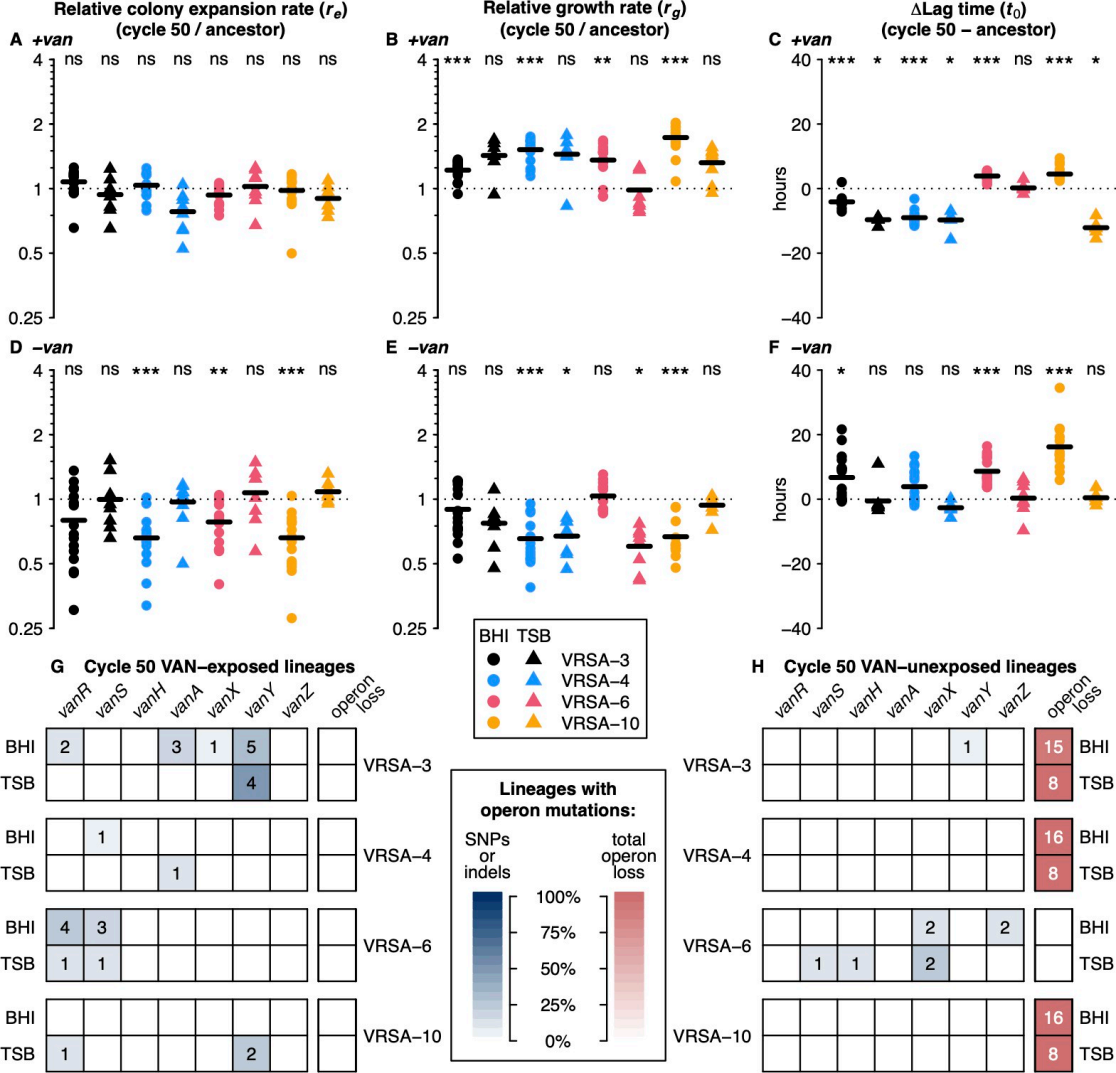
VAN-exposed lineages exhibited a fitness tradeoff in the presence and absence of vancomycin. (**A-F**) Shown are growth parameters of cycle 50 VAN-exposed lineages relative to ancestral VRSA strains (dotted horizontal lines), as measured in the presence (**A-C**; 32 μg/ml) and absence (**D-F**) of vancomycin. (**A** and **D**) Relative colony expansion rates (***r***_***e***_ evolved / ***r***_***e***_ ancestor) of cycle 50 VAN-exposed lineages on solid media revealed that colonies generally expanded more slowly in the absence of vancomycin after evolution, with little change in ***r***_***e***_ in the presence of vancomycin. Colony expansion rate was measured once per evolved lineage. (**B** and **E**) VAN-exposed lineages generally displayed faster growth rate (***r***_***g***_ evolved / ***r***_***g***_ ancestor > 1) in liquid in the presence of vancomycin after evolution, but slower growth in its absence. (**C** and **F**) Difference in lag time between ancestral and VAN-exposed lineages (***t***_***0***_ evolved–***t***_***0***_ ancestor) in the presence (32 μg/ml) and absence of vancomycin showed increased lag times in the absence of vancomycin and decreased lag time in the presence. For each evolved lineage, growth rate and lag time were calculated from the mean of 24 replicates. Each group of relative values was compared to 1 (relative growth rate/colony expansion rate) or 0 (Δ***t_0_***) by two-sided Wilcoxon signed-rank test and asterisks denote statistical significance after Holm-Bonferroni multiple testing correction (*** = p-adj. < 0.001, ** = p-adj. < 0.01, * = p-adj. < 0.05, ns = p-adj. ≥ 0.05). (**G** and **H**) Presence of mutations in *vanA* operon in VAN-exposed (**G**) and VAN-unexposed (**H**) lineages revealed mutations were common among VAN-exposed lineages. There was global loss of the *vanA* operon for most VAN-unexposed lineages. The number of lineages with ≥ 1 mutation in the indicated gene is printed in each box.

We hypothesized that propagation of VRSA in the presence of vancomycin would select for compensatory mutations that increase fitness. To this end, we compared the fitness of cycle 50 VAN-exposed lineages to that of their respective ancestral strains. Colony expansion rate on vancomycin-containing agar did not change relative to the ancestral strains (Fig 3A), but growth rate increased (Fig 3B) and lag time decreased (Fig 3C) in vancomycin for most VAN-exposed lineages. These results indicated that VRSA was able to adapt to growth in vancomycin. In contrast, colony expansion rate, growth rate, and lag time in the absence of vancomycin all displayed a fitness reduction in VAN-exposed lineages (Fig 3D–3F). Taken together, these results suggest a fitness trade-off between growth with and without the presence of vancomycin.

In parallel to VAN-exposed lineages, we propagated separate lineages of each VRSA strain in the absence of vancomycin to assess the role of adaptation to growth medium (Fig 1A). Colony expansion rate of VAN-unexposed lineages remained largely unchanged following evolution (S3A Fig). While there were statistically significant changes in growth rate and lag time in VAN-unexposed lineages, there was no directional trend observed across strains and the magnitude of these changes were small (S3B and S3C Fig), implying the fitness changes observed in VAN-exposed lineages were largely due to the presence of vancomycin.

We considered the possibility that mutations restoring fitness in the presence of vancomycin could have occurred within the *vanA* operon. Whole genome sequencing of all evolved lineages allowed identification of mutations enriched in VAN-exposed relative to VAN-unexposed lineages. We constructed a vancomycin-specificity score (see Materials and Methods) to detect mutations that occurred in response to *vanA* carriage or expression, rather than due to growth media or experimental setup (Fig 1A). Mutations in the *vanA* operon occurred in some VAN-exposed lineages, while global loss of the *vanA* operon and surrounding region occurred for most VAN-unexposed lineages (Figs 3G, 3H and S2). In the absence of vancomycin, lineages with mutations in the *vanA* operon had growth rates similar to their ancestral strains, while lineages without grew more slowly (two-sided Wilcoxon rank-sum p-value = 3.8E-3).

### Mutations in D-alanine:D-alanine ligase (*ddl*) are enriched in VAN-exposed lineages

**Fig 4:**
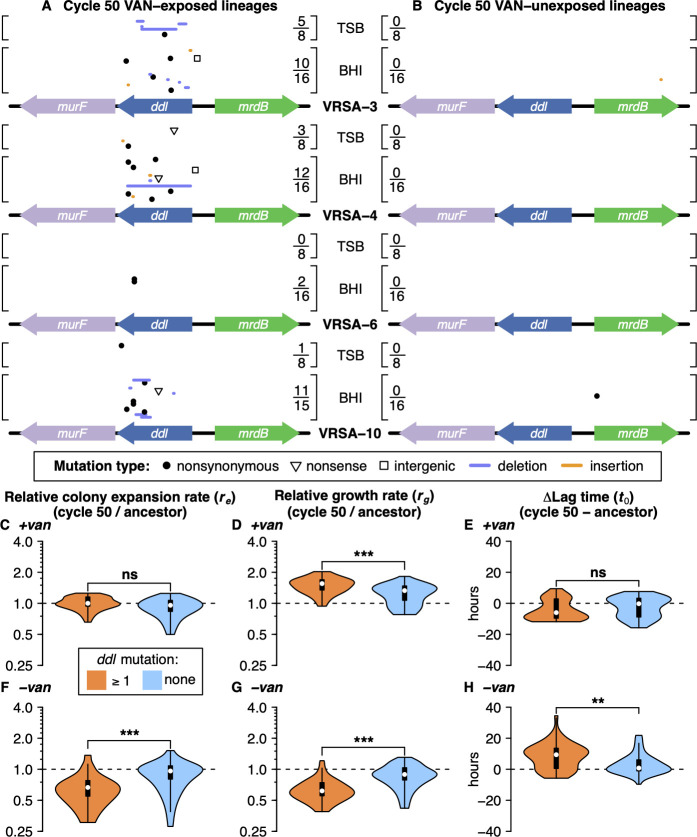
Widespread mutations in *ddl* in VAN-exposed lineages were associated with a fitness tradeoff. (**A-B**) Position and identity of mutations along *ddl* and the surrounding region in VAN-exposed (**A**) and VAN-unexposed (**B**) lineages. Mutations in independent lineages are separated vertically. Shown is the number of lineages with a mutation in *ddl* over the number of total cycle 50 lineages in each group. Numerous and varied mutations occurred within the *ddl* gene of VAN-exposed lineages, implying loss or partial loss of Ddl function was adaptive in the presence of vancomycin. (**C-H**) Shown are colony expansion rate, growth rate, and lag time relative to ancestral strains (horizontal dotted lines). (**C-E**) In the presence of vancomycin, lineages with a mutation in *ddl* (n = 44) had a faster growth rate than lineages without mutations in *ddl* (n = 51), but no statistically significant difference was seen in colony expansion rate or lag time between the groups. (**F-H**) In the absence of vancomycin, lineages with a mutation in *ddl* exhibited reduced colony expansion rate and growth rate, as well as longer lag times than lineages without mutations in *ddl*. The groups were compared by two-sided Wilcoxon rank-sum test. Asterisks denote statistical significance after Holm-Bonferroni multiple testing correction (*** = p-adj. < 0.001, ** = p-adj. < 0.01, * = p-adj. < 0.05, ns = p-adj. ≥ 0.05).

Many clinical isolates of VRSA and VRE have loss of function mutations in *ddl*, the gene encoding D-alanine:D-alanine ligase. The prevalence of *ddl* mutations in clinical isolates of VRSA and VRE suggests it is the target of compensatory mutations *in vivo*. Previous *ddl* mutations observed in VRSA isolates include nonsynonymous SNPs and a frameshift (S1 Table). Ancestral VRSA-6 used in this study harbors a *ddl* mutation resulting in a nonsynonymous substitution N308K that was found to decrease Ddl activity by 1000-fold [40]. Other VRSA strains also harbor *ddl* mutations (VRSA-8, -9, -11a, and -11b) resulting, in some cases, in a dependence upon vancomycin for growth, a tendency to maintain resistance in the absence of vancomycin, and greater sensitivity to oxacillin [37,39]. We hypothesized that VAN-exposed lineages of VRSA would harbor loss of function mutations in *ddl*. Ranking genes by vancomycin-specificity score revealed that mutations in *ddl* were enriched in VAN-exposed lineages and did not occur in VAN-unexposed lineages (Figs 4A, 4B, S4A and S4B). Mutations in *ddl* included nonsense mutations, large deletions, insertions, frameshifts, and nonsynonymous mutations (S1 Table). Single nucleotide substitutions in the intergenic space surrounding *ddl* were observed in two lineages. Interestingly, one BHI-propagated lineage of VRSA-10 acquired the same N308K mutation as in ancestral VRSA-6 (S1 Table). The vancomycin specificity and nature of the observed *ddl* mutations suggests that loss or partial loss of Ddl activity was adaptive in the presence of vancomycin. Interestingly, no genes other than *ddl* showed a statistically significant enrichment in mutations among cycle 50 VAN-exposed lineages (S4A Fig). Our results corroborate previous reports of *ddl*-inactivating mutations in clinical VRSA and VRE isolates and reinforce the applicability of our results to the evolution of VRSA in the clinic.

**Fig 5:**
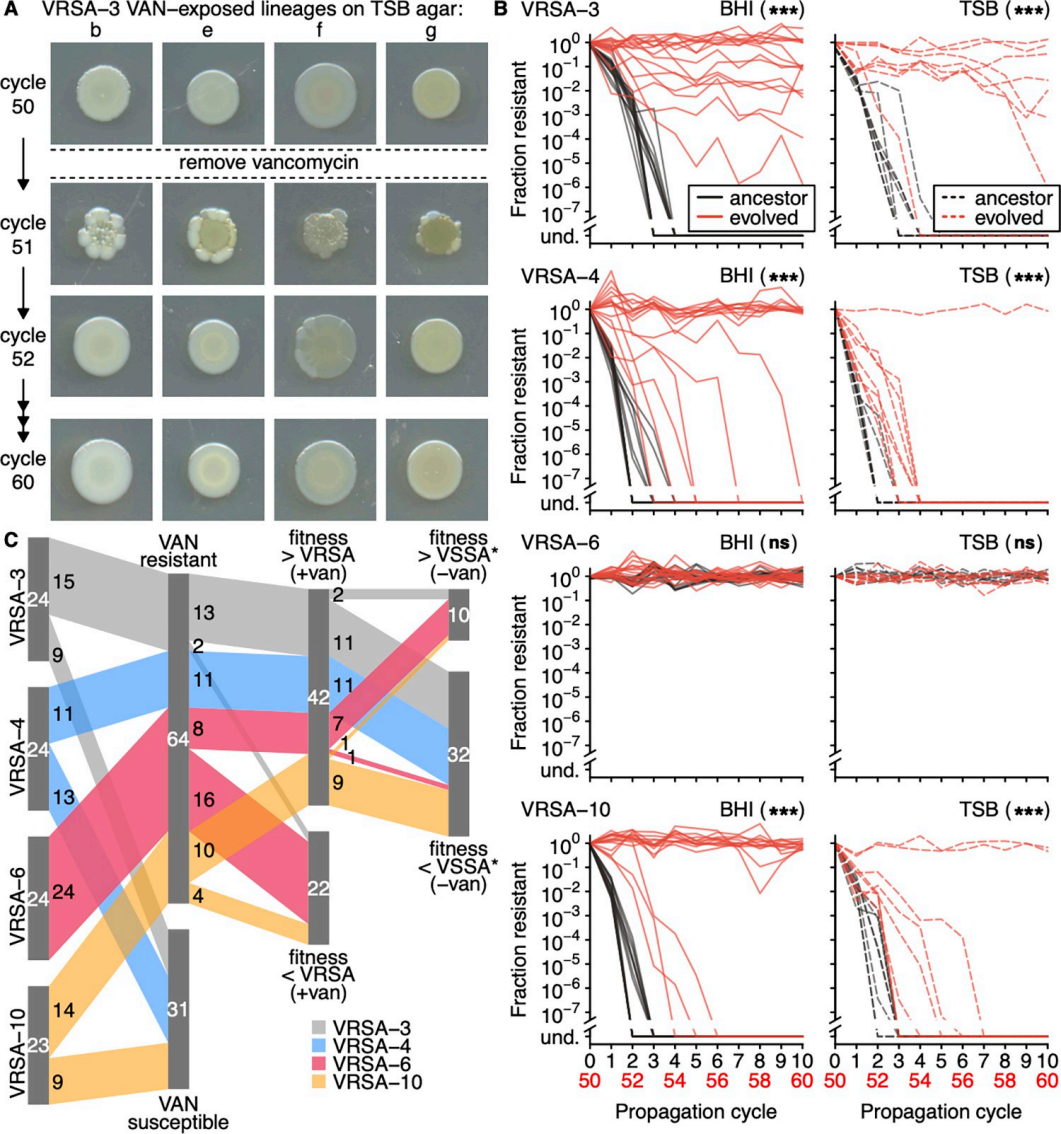
Many VAN-exposed lineages maintained resistance in the absence of vancomycin and exhibited abnormal colony morphology. (**A**) Many VAN-exposed lineages exhibited an abnormal colony morphology following the removal of vancomycin (at cycle 51) that was rapidly alleviated. Shown are representative VRSA-3 lineages after 66 hours of growth during cycles 50, 51, 52, and 60 (see also S6A Fig). Colony images were cropped and spliced from larger scans for clarity and to save room. Adjustments to brightness were applied uniformly to each cycle. (**B**) VAN-exposed lineages maintained resistance in the absence of vancomycin or reverted to susceptibility more slowly than their ancestral strains on both media (und. = no resistant cells detected). Reversion curves of the VAN-exposed lineages were compared with their respective ancestral strain by log-rank test and asterisks denote statistical significance after Holm-Bonferroni multiple testing correction (*** = p-adj. < 0.001, ** = p-adj. < 0.01, * = p-adj. < 0.05, ns = p-adj. ≥ 0.05). All VAN-exposed VRSA-6 lineages maintained resistance through propagation cycle 60. (**C**) Sankey diagram showing the number of lineages that overcame each fitness barrier. Many (64/95) lineages maintained resistance in the absence of vancomycin through cycle 60 (“VAN resistant”) and had greater fitness than their respective ancestral VRSA strain in the presence of vancomycin in two or more fitness proxies simultaneously (“fitness > VRSA (+van)”). Only a few (10/95) lineages also had greater fitness than their respective ancestral VSSA strain in the absence of vancomycin in two or more fitness proxies simultaneously (“fitness > VSSA (-van)”). * VRSA-6 evolved lineages were compared to ancestral VRSA-6 in the absence of vancomycin since an ancestral VSSA-6 was not isolated.

To determine if loss of function mutations in *ddl* were fitness-compensating, we compared the fitness of VAN-exposed lineages with mutations in *ddl* to those without and found that *ddl* mutations were associated with faster growth rate in the presence of vancomycin (Fig 4D) and poorer fitness in its absence (Fig 4F–4H). Some VAN-exposed lineages (27%) appeared vancomycin-dependent with long and variable lag times (i.e., ***t***_***0***_ mean > 15 h and ***t***_***0***_ standard deviation > 1.5 h) in replicate growth curves when grown without vancomycin (S5A–S5C Fig). Similarly, many VAN-exposed lineages (62%) exhibited abnormal colony morphology when grown on agar without vancomycin (Figs 5A and S6A). Abnormal colony morphology was associated with mutations in *ddl* (two-sided Fisher’s exact test p-value = 3.2E-9; S7A Fig). Such characteristics of vancomycin dependence are consistent with previous reports in VRSA and VRE isolates that harbor *ddl* mutations [37,39,41–43,56].

We measured the degree of complete vancomycin dependence and found that some lineages had only a small subpopulation of cells that were viable in the absence of vancomycin (S5D Fig). We used the proportion of viable cells to calibrate lag times using the viable inoculum size (S5F and S5G Fig). This revealed that lag times in the absence of vancomycin were much longer for VAN-exposed lineages than their respective ancestral VRSA strains (Fig 3F). Excluding VRSA-6 for which *ddl* mutations were rare, all lineages with a viable subpopulation in the absence of vancomycin smaller than 10% harbored a *ddl* mutation. While this experiment revealed phenotypic heterogeneity in some lineages, whole genome sequencing showed all *ddl* mutations had reached fixation (100% frequency) by propagation cycle 50. However, we hypothesized very low frequency alleles might be missed in sequencing due to insufficient coverage. To address this, where possible, we designed primers that would amplify wild type but not mutated *ddl* alleles (i.e., one or both primers were within a deleted region). We performed qPCR and found that some but not all lineages with a small fraction viable in the absence of vancomycin had late amplification of the wild type allele (S5E Fig), implying that the heterogeneity observed is due, in some cases, to incomplete purging of the wild type allele from the population.

### VAN-exposed lineages are less likely to revert to susceptibility in the absence of vancomycin

We hypothesized that VAN-exposed lineages would be less likely than ancestral strains to revert to susceptibility in the absence of vancomycin due to compensation for the cost of vancomycin resistance [46]. Following 50 propagation cycles in the presence of vancomycin, we propagated VAN-exposed lineages for 10 propagation cycles on vancomycin-free agar (Fig 1A). In contrast to their ancestral strains, many (67%) VAN-exposed lineages maintained resistance in the absence of vancomycin (Fig 5B and 5C). Some lineages did undergo reversion to susceptibility (33%), albeit generally at propagation cycles later than their respective ancestral VRSA strains. Interestingly, 12 VRSA-3 lineages partially reverted to susceptibility, with a subpopulation of resistant cells remaining (Fig 5C).

Whole genome sequencing revealed that 23% (10/44) of lineages with *ddl* mutations at cycle 50 harbored a wild type allele at cycle 60. Due to the improbability of reversion mutations and our evidence of low frequency wild type *ddl* clones at cycle 50 (S5E Fig), these results suggest that some lineages underwent “clone replacement” with a low frequency clone at cycle 50 that rose to dominance by cycle 60. We cannot rule out the possibility that other lineages underwent unobserved “clone replacement” events. Interestingly, a VAN-exposed lineage of VRSA-3 had a one base pair insertion in *ddl* at cycle 50 but restored the frame via a four base pair deletion before cycle 60, resulting in a one amino acid deletion (Q301) and I302L substitution relative to ancestral VRSA-3 (S1 Table and S7B Fig). This might be a case of “pseudogene repair” [57], but we did not investigate the function of the modified Ddl protein.

We hypothesized that lineages with mutations in *ddl* would be more likely to maintain resistance in the absence of vancomycin, because VRSA-6, which harbors a *ddl*-inactivating mutation, did not revert to vancomycin susceptibility. Since several VRSA-3 lineages underwent partial reversion to vancomycin susceptibility, we included them in the reversion group if the dominant clone appeared to be susceptible by phenotypic and sequencing analysis. Lineages with *ddl* mutations largely maintained resistance (S7C Fig; two-sided Fisher’s exact test p-value = 5.7E-11). We found only one lineage that reverted to susceptibility and harbored a mutation 43 base pairs upstream of *ddl* (S1 Table). This suggests that maintenance of vancomycin resistance is due to dependence on *vanA* for the synthesis of peptidoglycan rather than *ddl*.

### VRSA can escape the fitness tradeoff imposed by adaptation to growth in vancomycin

We assessed the fitness of cycle 60 lineages to determine if the fitness tradeoff observed at cycle 50 had been alleviated. We compared the fitness of evolved lineages with their respective ancestral VRSA strain in the presence of vancomycin and to the corresponding ancestral VSSA strain in its absence (Fig 5C). Since we did not isolate a vancomycin-susceptible ancestral strain of VRSA-6, we used the fitness of ancestral VRSA-6 in the absence of vancomycin for comparison. A substantial proportion (64/95) of lineages maintained resistance through cycle 60. Of these, 42 lineages had greater fitness than their respective ancestral VRSA strain in the presence of vancomycin in two or more fitness proxies simultaneously. Of this subset, only 10 had greater fitness than their respective VSSA strain in the absence of vancomycin in two or more fitness proxies simultaneously. This group was largely composed of VRSA-6 lineages (Fig 5C). Assuming the isolated VSSA was reflective of the original MRSA infection before the acquisition of vancomycin resistance, these results imply very few (10/95) VAN-exposed lineages adapted to overcome the cost of resistance according to these criteria.

### Some VAN-exposed lineages became oxacillin-susceptible

We measured the linezolid, oxacillin, vancomycin and sulfamethoxazole-trimethoprim (SXT) MICs of ancestral strains and evolved lineages using a modified broth microdilution method in which we substituted cation-adjusted Mueller-Hinton Broth (MHB) for BHI or TSB to match the propagation conditions of the evolved lineages. Vancomycin MICs remained largely unchanged in VAN-exposed lineages, as well as most VAN-unexposed lineages of VRSA-6 (S8A and S8B Fig). As expected, vancomycin MICs of VAN-unexposed lineages decreased to below the clinical breakpoint in all lineages of VRSA-3, -4, and -10, as well as one lineage of VRSA-6 (S8B Fig). The SXT MICs of evolved lineages remained largely unchanged in BHI-propagated lineages but decreased substantially in TSB-propagated lineages of VRSA-3, -4, and -10 (S8C and S8D Fig). A majority of evolved lineages lost oxacillin resistance or had a significant decrease in oxacillin MIC relative to ancestral strains (S8E and S8F Fig). VAN-exposed lineages with a mutation in *ddl* had lower oxacillin MICs than those without (two-sided Wilcoxon rank-sum test p-value = 8.8E-3). These results are consistent with previous work showing vancomycin-dependent VRSA-6, -8, and -11 exhibited decreased oxacillin resistance due to the production of peptidoglycan precursors ending in D-alanyl-D-lactate, which are incompatible with penicillin-binding protein PBP2A that confers resistance to β-lactams [58]. However, the relationship between *ddl* mutations and oxacillin resistance was abolished by cycle 60 (two-sided Wilcoxon rank-sum test p-value = 0.55), in part due to increases in oxacillin resistance that might be related to the restoration of wild type *ddl*.

Although some VAN-exposed lineages remained oxacillin-susceptible after propagation in the absence of vancomycin (i.e., cycles 51–60; S9B Fig), oxacillin MICs generally increased between cycle 50 and 60 (two-sided Wilcoxon signed-rank test p-value = 0.023). Oxacillin susceptibility presents a potential opportunity to provide oxacillin therapy in the case that a strain of VRSA begins to spread in the clinic. However, of the 10 lineages that overcame all fitness barriers according to our criteria (Fig 5C), five were still resistant to oxacillin. Relatedly, linezolid MICs remained largely unchanged after propagation (Fig S8H and S8G). Continued susceptibility to linezolid may initially curtail the spread of a fit VRSA strain in the clinic, although we believe that VRSA might be able to overcome all antibiotics tested here given enough opportunities. Further work is required to assess the threat of an *S*. *aureus* strain resistant to all antibiotics of last resort.

## Discussion

The fitness cost imposed by *vanA*-mediated resistance has been proposed as one of the barriers preventing an epidemic of VRSA. This fitness cost manifests as slower growth and longer lag time in the presence of vancomycin and the instability of resistance in the absence of vancomycin [36]. By experimentally evolving replicate lineages of four clinical VRSA strains, we measured the potential of this pathogen to overcome the fitness cost of resistance. VAN-exposed lineages grew faster with a shorter lag time on vancomycin but appeared less fit in the absence of vancomycin. Associated with this fitness trade-off were widespread mutations in *ddl*, which was previously implicated in the development of vancomycin dependence in clinical isolates of VRSA and VRE. The function of Ddl is substituted by VanA under vancomycin induction, thus loss-of-function mutations may occur readily in the presence of vancomycin. Our study suggests *ddl* mutation is the primary means by which VRSA is able to alleviate the fitness cost imposed by *vanA*-mediated resistance, but *ddl* mutations impose a fitness deficit in the absence of vancomycin. Further propagation without vancomycin allowed only a handful of VAN-exposed lineages to escape the fitness tradeoff and overcome the cost of resistance while maintaining a functional *vanA* operon.

In contrast to their ancestral VRSA strains, many VAN-exposed lineages maintained vancomycin resistance following 10 propagation cycles in the absence of vancomycin. Nearly all lineages that maintained resistance through cycle 60 had a mutation in *ddl*, suggesting a newfound dependence on the *vanA* operon for peptidoglycan synthesis. Hence, the loss of *ddl* represents a compensatory mutation that impedes the loss of vancomycin resistance upon drug removal. Our results imply the durability of vancomycin in *S*. *aureus* is due to the number of fitness tradeoffs that occur during compensatory evolution, including increased fitness in the presence of vancomycin and decreased fitness in its absence, as well as growth in the presence of oxacillin. However, we maintain that there is a clinical risk associated with the potential for spread of *vanA*-mediated vancomycin resistance and not all these vancomycin-resistant strains will be susceptible to oxacillin. Thus, we cannot rule out the possibility *S*. *aureus* could evolve resistance to all antibiotics of last resort based on its infamous historical ability to acquire antibiotic resistance.

Most ancestral strains rapidly reverted to susceptibility in the absence of vancomycin, suggesting maintenance of the plasmid bears a large fitness cost. This cost could be the result of harboring two competing mechanisms for cell wall synthesis, except that *vanA* expression is induced only by the presence of glycopeptides [59]. Therefore, the fitness cost could be due to leaky expression of the *vanA* operon, and it is known that VanX and VanY exhibit activity in the absence of induction [35,40]. Our results demonstrate that continued vancomycin exposure selects for compensatory adaptations that alleviate this fitness cost in the presence of vancomycin and that further adaptations can compensate for the fitness cost in the absence of vancomycin. Treatment failure due to VRSA would necessitate discontinuation of vancomycin therapy because of its inefficacy, as well as to prevent adaptation to growth in the presence of the drug. Such a strategy has the potential to prolong the lifetime of the drug by evading the evolution of low-cost resistance.

VRSA-6 differed from the other three strains in that, prior to experimental propagation, it harbored a nonsynonymous mutation in *ddl*, leading to the N308K substitution. This mutation was shown to reduce the activity of Ddl by ~1000-fold [40], which likely caused VRSA-6 to maintain resistance in the absence of vancomycin and contributed to its decreased oxacillin MIC. Additionally, VRSA-6 grew slower than all of the other strains in the absence of vancomycin and exhibited superior growth in its presence. Despite having possibly adapted to growth in vancomycin, VRSA-6 was not observed to spread between patients. There are many barriers to patient transmission, including the site of infection, transmission bottlenecks, pathogen fitness and virulence, and random chance. Although VRSA-6 overcame the fitness deficit imposed by *vanA*-mediated resistance, this was insufficient to ensure transmission. The lack of spread of VRSA is not particularly surprising considering many infections do not transmit between patients. We suspect it could be a matter of time before a VRSA strain is able to overcome transmission barriers and proliferate, as was the case for MRSA [60].

Interestingly, VRSA-3, -4, and -10 rapidly reverted to susceptibility in the absence of vancomycin on solid medium when the colony perimeter was transferred but maintained resistant subpopulations in liquid medium and on solid medium when the whole colony was transferred (Fig 2G–2I). This discrepancy highlights the impact of solid versus liquid substrates in evolution experiments, which have traditionally been carried out solely in liquid media. Growth dynamics on solid medium differ from traditional exponential growth and can yield unexpected evolutionary outcomes [61], which may more reliably mimic *in vivo* growth (e.g., biofilms). Colony growth may yield more mutations than in well-mixed liquid culture due to the process of "allele surfing" at the expanding front of colonies [62]. Unexpectedly, in VAN-exposed lineages, colony expansion rates did not always track increases in growth rate following propagation on agar media. This result implied either a limitation in colony expansion rate or changes in colony thickness that were unmeasured.

In this work, we showed significant differences in measures of fitness between ancestral VRSA strains and evolved lineages. However, we did not perform competition experiments between evolved and ancestral strains to corroborate these findings due to the magnitude of this experiment (i.e., 191 evolved lineages). Competition experiments are considered the gold standard for assessing fitness differences as they encompass multiple parameters of fitness including lag time, growth rate, resource utilization efficiency, yield, and die off in stationary phase [63]. Individual measures of fitness such as growth rate in monoculture are imperfect proxies for fitness, as strains with the same or similar growth rate in isolation may outcompete each other in competition by the production of an inhibitory molecule or the ability to more effectively metabolize a limiting resource [63]. Despite this, exponential growth rate is commonly used in adaptive laboratory evolution experiments as a proxy for fitness [64–66]. To overcome the limitations of assessing fitness by exponential growth rate alone, we also measured lag time in liquid media and colony expansion rate on agar media.

Importantly, fitness proxies do not indicate the virulence potential of a laboratory-derived strain which requires *in vivo* measurements that we did not perform. Other limitations of our study included the use of short-read sequencing that may prevent the identification of some mutations (e.g., gene duplications). Our sequencing protocol yielded 25-30x coverage, precluding the identification of low frequency alleles that may have been present in the ‘meta-population’ of each lineage. This limitation was clear as the dominant clone in some cycle 60 lineages with wild type *ddl* was likely present at cycle 50, but not sequenced. Additionally, we sometimes measured MICs in BHI and TSB rather than cation-adjusted MHB as is standard for broth microdilution. However, MICs of ancestral strains measured by broth microdilution in MHB medium did not substantially differ from BHI and TSB in most cases (S8 Fig). VRSA-3, -4, and -10 appeared resistant to sulfamethoxazole-trimethoprim when measured in BHI and TSB, but not MHB. The reason for this discrepancy was unknown.

Several unexplained results are presented in this manuscript and represent limitations beyond those mentioned previously. First, a VAN-unexposed lineage of VRSA-3 (3c_BHI) reverted to vancomycin susceptibility early in the propagation experiments. However, unlike other lineages, Illumina sequencing showed high coverage of the contig on which the *vanA* operon resides. This lineage harbored a tandem repeat expansion in *vanY*. Interestingly, an identical mutation was observed in several VAN-exposed lineages of VRSA-3 (S2 Fig), suggesting this mutation may be the result of sequencing/assembly errors. The basis of vancomycin susceptibility in this lineage is unclear. Second, for an unknown reason, a VAN-exposed lineage of VRSA-10 (10p_BHI) failed to grow on cycle 21, even after repeated attempts to grow it from the previous propagation cycle. Finally, we cannot rule out the possibility of cross-well contamination in resuspension plates. Lineages of the same strain, media, and vancomycin condition were held in adjacent wells in 96-well plates. We suspected that Illumina sequencing would reveal near identical mutation profiles in such cases. To this end, we calculated the Jaccard similarity of mutation lists between each pair of lineages in each strain-media-condition group. Two lineages of VAN-exposed VRSA-10 (10f_BHI and 10g_BHI) showed a high proportion of shared mutations at cycle 50, including the same mutation in *ddl* (S1 Table). Interestingly, this mutational similarity was not present at cycle 60 (including loss of the *ddl* mutation in 10f_BHI). No other lineages showed such a relationship in this analysis.

Since many antibiotic resistance mechanisms impose a fitness cost, it is often necessary to subculture samples of clinical isolates in the presence of antibiotic to maintain laboratory stocks. Inherently, this process selects for mutations that compensate for the cost of resistance and may increase the fitness of the strains being propagated. Our study highlights the potential adaptations made by VRSA during simple laboratory passaging in the presence of vancomycin to maintain resistance, a process being performed in many labs working with this BSL-2 organism. The frequency of this occurrence further reinforces the importance of work aimed at understanding the implications of continued growth of clinical isolates with antibiotic resistances. Here we took significant precautions to ensure the safety of our work. It is important that labs propagating their microorganism of interest are sufficiently considering the safety implications of evolution during standard laboratory culturing.

Our results indicate the durability of vancomycin against *S*. *aureus* is likely due to the fitness tradeoff that occurs during adaptation to the presence of vancomycin. Based on these results, it is plausible for a high-fitness VRSA strain to emerge that is able to maintain resistance in the absence of drug exposure. Such an outcome would be a major concern, as vancomycin is the primary treatment for MRSA and few alternative antibiotics exist. Therefore, we believe it is well-justified to continue the development of alternative treatments for MRSA, including vancomycin analogs [67], new antibiotics [68], antimicrobial peptides [69], phages [70,71], and other alternatives to antibiotics [72]. Vancomycin has, thus far, remained remarkably durable, but the results of this study imply that prolonged durability cannot be taken for granted.

## Materials and methods

### VRSA strain isolation

Fourteen VRSA strains available from the Biodefense and Emerging Infections Research Resources Repository were obtained with Institutional Biosafety Committee approvals for experiments. VRSA-3a, VRSA-4, VRSA-6, and VRSA-10 were chosen for subsequent experiments based on their susceptibility to other antibiotics (linezolid and sulfamethoxazole-trimethoprim). Frozen stock solutions were streaked onto 1.5% agar containing Bacto Brain Heart Infusion (BHI) medium with 32 μg/ml vancomycin and incubated at 37°C for 48 h. A single colony was inoculated into 4 ml BHI broth (with 32 μg/ml vancomycin) and incubated at 37°C for 24 h with orbital shaking at 200 rpm. Liquid cultures were centrifuged (2400 rcf for 4 min) and pellets resuspended in 4 ml 60% glycerol (~5x10^5^ colony forming units [CFUs]/μl). Stocks of ancestral strains were maintained at -80°C.

### Safety procedures and long-term strain storage

All VRSA strains used in this study are classified as biosafety level 2. Due to the potential risk of human exposure to the strains used in this study, institutional approval was obtained from the Institutional Biosafety Committee of the University of Pittsburgh prior to the work (IBC201700232 & IBC201800007), and responsible biosafety procedures were followed as outlined below. All lab personnel working with VRSA successfully completed safety training provided by the University of Pittsburgh’s Environmental Health and Safety Office. Personal protective equipment (PPE) was worn at all times when working with VRSA strains, including gloves, lab coat, and eye protection. Aerosol-barrier pipet tips were used exclusively while working with VRSA strains. All work done with VRSA strains was performed in a biosafety cabinet which was UV-decontaminated for 15 min after each use. Biohazard waste (i.e., pipet tips, microcentrifuge tubes, and gloves) generated during use with VRSA was collected in a covered container and autoclaved prior to disposal according to University biohazard protocols. Incubators in which VRSA samples were being grown were labeled appropriately. Glass test tubes of liquid culture were decontaminated using 50% bleach (1:1 volume) for a minimum exposure of 4 h. VRSA samples in freezer storage (-80°C) were stored on a separate shelf from all other laboratory samples. 96-well plates were sealed with an aluminum seal and placed into a freezer box. Freezer boxes containing VRSA samples were labeled appropriately, and an inventory of freezer box contents was maintained and made available to all laboratory members. Freezer storage was labeled with contact information of authorized personnel in case of power failure.

### Experimental evolution by propagation

Each strain was propagated on two media in the presence (32 μg/ml) and absence of vancomycin, including 16 replicate lineages in BHI and 8 replicate lineages in Tryptic Soy Broth without dextrose (TSB). Two μl of ancestral stocks were deposited onto the center of 60 mm petri dishes containing 15 ml of 1.5% agar. Drops were allowed to dry before sealing with parafilm and laying top-down on flatbed scanners in a 37°C incubator for ~70 h. Plates were scanned at 400 dpi every 6 h. After ~70 h of growth, a 10 μl pipette tip was used to gather the outer edge of each colony (~10^9^ of ~10^10^ CFUs per colony), which was deposited into 100 μl of 60% glycerol in a 96-well plate. These resuspension plates were used to inoculate the next propagation cycle with 2 μl (~10^7^ CFUs) of the resuspension solution. Therefore, each propagation cycle corresponded to at least 10 generations of growth. This process was repeated for 50 propagation cycles. The cycle 50 VAN-exposed lineages were propagated for a further 10 cycles on vancomycin-free agar with the same procedure as described above.

In order to preserve cycle 50 and 60 lineages, overnight cultures were made by inoculating 10 μl from each well of the resuspension plates into 4 ml of matching growth medium with (32 μg/ml) or without vancomycin and incubated at 37°C for 24 h with orbital shaking at 200 rpm. Liquid cultures were centrifuged (2400 rcf for 4 min) and pellets resuspended in 4 ml 60% glycerol at a concentration of ~5x10^5^ CFUs/μl. These stock solutions were frozen at -80°C.

### Passaging in liquid medium

Each strain was passaged in liquid media (BHI and TSB) without vancomycin in three, independent replicates. Five μl of ancestral stocks were added to 5 ml of liquid medium in glass test tubes which were incubated for 24 h at 37°C with orbital shaking at 200 rpm. From each overnight tube, 5 μl was used to inoculate the next passaging cycle. These transfer volumes were selected to match the initial and final population sizes on solid medium. In total, 10 passaging cycles were completed.

### Propagating whole colonies on agar

Each strain was propagated on solid media (BHI and TSB) without vancomycin in three, independent replicates. Two μl of ancestral stocks were pipetted onto the center of 60 mm petri dishes containing 15 ml of 1.5% agar. Drops were allowed to dry before sealing with parafilm and laying top-down in a 37°C incubator for 18 h. Whole colonies were resuspended in 200 μl 60% glycerol, which was diluted 10-fold and used to inoculate the next propagation cycle. In total, 10 propagation cycles were completed.

### Reversion to susceptibility

To measure reversion to susceptibility, resuspension plates were thawed and each well was serially diluted in 10-fold steps. Each dilution was plated onto agar medium with (32 μg/ml) and without vancomycin and CFUs were counted after 24 h incubation at 37°C. Thus, our definition of reversion to susceptibility refers to the inability to grow on agar medium (BHI or TSB) that contains 32 μg/ml vancomycin. The fraction resistant was calculated using the following equation:

$$Fraction\;resistant=\frac{{CFUs}_{\;32\;\mu g/ml\;vancomycin\;agar}}{{CFUs}_{agar}}$$

### Colony expansion rate measurements

Colony area was measured from colony scans using ImageJ [73] and the colony radius was calculated assuming circularity. Expansion rate was extracted from a linear model fit to the radius and time data points from 24–66 h (e.g., see Fig 1C).

### Determination of growth rate and lag time

Growth rate was measured using optical density at 600 nm (OD_600_) collected at 10 to 15 min intervals over 48 h. Stocks were thawed and diluted 10-fold in growth medium, then 1 μl was inoculated into each well of a 96-well plate containing 199 μl medium (i.e., 1:2000 total dilution). Plates were sealed with a gas-permeable membrane (Breathe-Easy, RPI), incubated at 37°C for 48 h and were then read with a Biotek Epoch II Microplate Spectrophotometer connected to a Biotek Biostack II (Agilent). Absorbance readings were made after 15 sec of fast orbital shaking at 307 rpm. Growth measurements were replicated across wells for each ancestral strain (n = 96) and evolved lineage (n = 24). The growth rate was calculated from the OD_600_ points using the following equation (MA_4_ = moving average with window size of four time points):

$$r_{g}={max(MA}_{4}(\frac{\Delta \log\left({OD}_{600}\right)}{\Delta time}))$$

We assumed exponential growth up to the time when growth rate was measured:

$$N_{1}=N_{0}e^{r_{g}(t_{1}-t_{0})}$$

This allowed lag time to be estimated from OD_600_ vs. time data using the following equation (e.g., see Fig 1C):

$$t_{0}=t_{1}-\frac{\ln\left(\frac{N_{1}}{N_{0}}\right)}{r_{g}}$$

where ***t*_**0**_** is lag time, ***t*_**1**_** is time when OD_600_ is 0.05 above background, ***N***_**1**_ is number of cells at time ***t*_**1**_** based on an approximate conversion of 8x10^5^ CFUs at this OD_600_, ***N***_**0**_ is the estimated inoculum size (1x10^5^ CFUs; from CFU plating measurements of overnight cultures), and ***r***_***g***_ is growth rate calculated above.

### Statistical tests

To compare colony expansion rate (***r***_***e***_; n = 8 for TSB or n = 16 for BHI), growth rate (***r***_***g***_; n = 96), and lag time (***t***_***0***_; n = 96) of ancestral VRSA strains in the presence and absence of vancomycin, we performed a two-sided Wilcoxon rank-sum test (*wilcox*.*test*) using R (v4.2.3) [74]. To compare fitness measures of ancestral VRSA strains and evolved lineages on a group-by-group basis (e.g., VAN-exposed VRSA-3 propagated on TSB), we performed a two-sided Wilcoxon rank-sum test of the means of evolved lineages (n = 8 TSB lineages or n = 16 BHI lineages) against the mean of the respective ancestral strain. To compare cycle 50 and cycle 60 VAN-exposed fitness measures, we performed a two-sided Wilcoxon signed-rank test for each group. P-values of statistical tests were corrected for multiple comparisons on a panel-by-panel basis using the Holm-Bonferroni method.

Reversion to susceptibility of ancestral VRSA strains and VAN-exposed lineages were considered as survival curves and compared by log-rank test. Type I censoring was present in both cases as the censoring time was fixed for all groups at the 10^th^ propagation cycle. The survival time of censored individuals was imputed as being equal to the censoring time to conservatively bias toward underestimating survival time. We compared survival curves of each group (e.g., VRSA-3 on TSB agar) with the log-rank test in R (v4.2.3) [74] using the *survdiff* function which is part of the *survival* package (v3.5–5) [75]. P-values of the eight log-rank tests were adjusted by the Holm-Bonferroni method to correct for multiple comparisons.

### Antibiotic minimum inhibitory concentration (MIC) determination

MIC measurements were conducted in triplicate in three consecutive wells of a 96 well plate, using OD_600_ as a proxy for growth. Wells containing 199 μl media and antibiotic were inoculated with 1 μl of a 2-fold dilution of cycle 50 stocks (~2.5x10^5^ CFUs) in growth medium matched to the propagation conditions. Plates were incubated at 37°C on BT Lab Systems Microplate Shaker, with constant shaking at 200 rpm. OD_600_ readings were taken at 48 h using a Biotek Epoch II Microplate Spectrophotometer. Wells were considered grown if OD_600_ was ≥ 0.05 above background at 48 h. MICs were defined as the concentration above where growth was observed in at least two out of three replicate wells. MICs were determined for vancomycin (0.125–2048 μg/ml), oxacillin (0.5–512 μg/ml), linezolid (0.03125–16 μg/ml), and sulfamethoxazole-trimethoprim (0.5/9.5-16/304 μg/ml) in 2-fold increments.

Stock solutions of ancestral strains were used to inoculate cation-adjusted Mueller-Hinton Broth (MHB). After overnight growth, each culture was diluted in MHB to a final concentration of ~5x10^7^ CFUs/ml. We added 1 μl of the diluted overnight culture to 99 μl of MHB and antibiotic for a final concentration of ~5x10^5^ CFUs/ml (0.5 McFarland turbidity standard). Plates were incubated and read as described above. Broth microdilution MIC was determined for vancomycin, oxacillin, linezolid, and sulfamethoxazole-trimethoprim at the same concentrations as above (S8 Fig).

### Dilution to extinction experiments

Stock solutions of each cycle 50 evolved lineage were thawed and serially diluted in 10-fold steps to 10^−6^. In 96-well plates, 1 μl of each dilution was added to 199 μl media. Each dilution was used to inoculate 24 wells containing liquid medium with vancomycin (32 μg/ml) and 24 wells containing vancomycin-free liquid medium. Growth medium (BHI or TSB) matched the propagation conditions of the evolved lineage. Plates were sealed with BreatheEasy membranes, shaken at 200 rpm, and incubated at 37°C for 96 h. Wells were considered grown if their OD_600_ was ≥ 0.05 above background at 96 h. Population sizes in each condition (+van/-van) were calculated in the same way as CFU counts. Relative population size in the absence of vancomycin is shown in S5 Fig.

### DNA sequencing and mutation identification

DNA sequencing was performed as described previously [76]. Briefly, genomic DNA was isolated from ancestral strains and evolved lineages using DNeasy Blood & Tissue Kit (Qiagen) with 4 μl of 2.5 mg/ml lysostaphin (Sigma) added to aid lysis. The concentration and purity of isolated DNA was measured on a NanoDrop 2000c spectrophotometer and samples were purified if A_260_/A_280_ ratio was not within 1.7–2.0, using the DNA Clean and Concentrate kit (Zymo Research). All samples were diluted to 2 ng/μl in nuclease-free water. DNA was fragmented and tagged using the Illumina DNA Prep Tagmentation kit. DNA libraries were amplified by PCR using the Q5 High Fidelity Polymerase in 50 μl reactions during which fragments were barcoded and multiplexed with custom i5 and i7 sequencing indexes. Barcoding PCR reactions were pooled and right and left-side size selection was performed on each pool using AMPure Sample Purification Beads (Beckman Coulter). The pooled libraries were sequenced by the Health Sciences Sequencing Core at the University of Pittsburgh Medical Center on the Illumina NextSeq 500 (cycle 50 library) or NextSeq 2000 (cycle 60 library) platform with 150 base pair single-end reads. All reads were deposited on the SRA (BioProject accession number PRJNA982761).

Reads were trimmed based on average quality greater than Q30. Reads from ancestral VRSA strains were assembled by the SPAdes Genome Assembler [77]. The program breseq (version 0.37.1) was run with default settings (non-polymorphism mode) to map reads of evolved lineages to the corresponding ancestral strain genome and generate a list of mutations [78]. We removed mutations that occurred on contigs < 500 base pairs in length. We also mapped reads from ancestral strains to the corresponding ancestral assembled genome and removed these mutations from evolved lineages. We were interested in genes that were vancomycin-specific and thus created a score to assess vancomycin specificity:

$${Vancomycin\;specificity\;score}_{gene}={log}_{e}\left(\frac{n_{VAN-exposed\;w/mutation}+1}{n_{VAN-exposed\;WT}+1}\right)-{log}_{e}\left(\frac{n_{VAN-unexposed\;w/mutation}+1}{n_{VAN-unexposed\;WT}+1}\right)$$

This scoring approach is equivalent to the log of the odds-ratio with pseudocounts added to handle cases where no lineages had a mutation in a given gene. For example, mutations in *ddl* occurred in 44/95 VAN-exposed lineages and in 0/96 VAN-unexposed lineage. The vancomycin specificity score for *ddl* is thus the log of: ((44+1) * ((96–0) + 1)) / (((95–44) + 1) * (0 + 1)). Fisher’s exact test (*fisher*.*test* in R) was used to calculate a p-value for each gene using the number of lineages in each group (i.e., VAN-exposed and VAN-unexposed) that harbored ≥ 1 mutation (*i*.*e*., p-values were calculated from raw counts without adding pseudocounts). S4 Fig shows the vancomycin specificity scores for each gene and its associated p-value. The VAN-exposed group used mutations data from either cycle 50 or cycle 60, shown in S4 Fig.

### Relative *vanA* copy number

Primer sets were designed to amplify a 112 base pair region of *vanA* and a 113 base pair region of the single-copy housekeeping gene *aroE* (Table 1). Standard curves were constructed for each primer set to estimate their amplification efficiency (*eff*). We performed qPCR in triplicate to get cycle threshold (Ct) values for each sample for each primer set using a QuantStudio 3 Real-Time PCR Thermocycler (Applied Biosystems). The following cycling conditions were used: 50°C– 2 min, 95°C– 3 min, and 40 cycles of 95°C—30 s, 54°C– 30 s, and 72°C– 30 s. To calculate relative *vanA* copy number, we used the equation:

$$\frac{{\left(1+{eff}_{vanA}\right)}^{{Ct}_{vanA}}}{{\left(1+{eff}_{aroE}\right)}^{{Ct}_{aroE}}}$$

**Table 1:** Primers used in this study.

Primer name	Sequence (5’– 3’)
*vanA* (forward)	TGCTCAGAGGAGCATGACGTAT
*vanA* (reverse)	TCCATACACCAGATTTCGTAATTCCA
*aroE* (forward)	ATGTTGATGAACAAGCGATTAATGCA
*aroE* (reverse)	GCTGTGCAATCCTTTAACATAACCAA
*ddl*– 3b_BHI (forward)	TCGATTCTGACAGCACAAAATGTA
*ddl*– 3b_BHI (reverse)	GCCTCTCCATTTTCTAAATGAAGCTC
*ddl*– 3e_BHI (forward)	AATGATGGTGATTGGAGAAAGCA
*ddl*– 3e_BHI (reverse)	CACCATTTCCTACATATGGT
*ddl*– 3d_TSB (forward)	ACCATACGATGCAGTATTCCCATTA
*ddl*– 3d_TSB (reverse)	TCCATAGAACTTGCAGCTGACAAT
*ddl*– 3f_TSB (forward)	CCAATGATGGTGATTGGAGAAAGC
*ddl*– 3f_TSB (reverse)	ACTGCATCGTATGGTTGTCCTG
*ddl*– 3g_TSB (forward)	CCTGCTAACTTAGGGTCAAGTGTA
*ddl*– 3g_TSB (reverse)	TCACCTGGCCATGTCGC

### Detecting ancestral *ddl* in evolved lineages

Primer sets were designed to amplify 100–200 base pair regions of *ddl* in five VAN-exposed lineages that harbored deletions (Table 1). We performed qPCR in duplicate for each evolved lineage. Each primer set was also used to amplify the ancestral strain DNA as a positive control. Primers designed to amplify a 113 base pair region of *aroE* were used as a positive control for genomic DNA (Table 1). PCR conditions were identical to those described above for amplifying *vanA*.

## Supporting information

S1 Fig Reversion to susceptibility was consistent with decreasing relative *vanA* copy number. One sample of each strain was propagated on BHI agar (0 μg/ml vancomycin) for 5 propagation cycles. As described in Materials and Methods, the perimeter of the colonies was harvested and we measured the fraction of cells that retained resistance and the relative copy number of the *vanA* gene (relative to *aroE*, see Materials and Methods). ‘C1’, ‘C2’, etc. refers to propagation cycle 1, 2, etc.
(TIFF)

S2 Fig Mutations in the *vanA* operon of evolved lineages. Mutations in the *vanA* operon and flanking gene(s) in cycle 50 VAN-unexposed (**A**) and VAN-exposed (**B**) lineages. (**A**) Most Cycle 50 VAN-unexposed lineages of VRSA-3, -4, and -10 show loss of the entire contig on which the *vanA* operon resides, consistent with their loss of resistance. A VRSA-3 lineage maintained the *vanA* operon, but had a 12 bp insertion in *vanY* and was susceptible to vancomycin. VRSA-6 maintained resistance in all but one BHI-propagated lineage which harbored a nonsense mutation in *vanX*. Some VRSA-6 lineages had mutations in some *vanA* operon genes. (**B**) Numerous and varied mutations were seen in cycle 50 VAN-exposed lineages throughout the *vanA* operon in some strains. Several genes appeared to have mutations across multiple lineages, including *vanR* and *vanS* in VRSA-6.
(TIFF)

S3 Fig The fitness proxies of VAN-unexposed lineages were similar to ancestral VRSA strains. Fitness changes in VAN-unexposed lineages did not follow a trend in either direction in colony expansion rate (**A**), growth rate (**B**), and lag time (**C**). Colony expansion rate was measured once per evolved lineage, while growth rate and lag time were taken from the mean of 24 technical replicates (see Materials and Methods). Each group was compared to the mean of the corresponding ancestral strain by a two-sided Wilcoxon signed-rank test. Asterisks denote statistical significance after Holm-Bonferroni multiple testing correction on a panel-by-panel basis (*** = p-adj. < 0.001, ** = p-adj. < 0.01, * = p-adj. < 0.05, ns = p-adj. ≥ 0.05).
(TIFF)

S4 Fig D-alanine:D-alanine ligase (*ddl*) mutations were enriched in VAN-exposed lineages. Genes were ranked according to their vancomycin specificity score (see Materials and Methods) using mutation data of evolved lineages at cycle 50 (**A-B**) and cycle 60 (**C-D**). (**A**) Volcano plot showing the vancomycin specificity score and its statistical significance for each gene at cycle 50. (**B**) A scatter plot showing the proportion of each group that harbored at least one mutation in each gene. Genes in and around the *vanA* operon appeared in the upper left corner (i.e., they occurred in VAN-unexposed lineages more often) due to segregational loss of the *vanA* plasmid and *ddl* in the lower right. (**C**) Relative to cycle 50, genes of the *vanA* operon have shifted down and to the right due to plasmid loss in several lineages that reverted to susceptibility or due to the acquisition of point mutations in lineages that retained resistance through cycle 60. Finally, *ddl* has shifted left and down relative to cycle 50 due to several lineages that apparently “reverted” to wild type *ddl* by cycle 60. (**D**) Relative to cycle 50, genes of the *vanA* operon have shifted to the right and *ddl* to the left for the same reasons as described previously. *SpA* refers to the gene staphylococcal protein A. P-values were calculated using Fisher’s exact test (two-sided *fisher*.*test*) for a difference in the proportions of each group that had at least one mutation in a gene. The p-value cutoff was calculated using the Bonferroni procedure considering the number of possible genes that were tested (n = 2,046). The single italicized letters refer to the genes of the *vanA* operon (i.e., *vanRSHAXYZ*).
(TIFF)

S5 Fig Some VAN-exposed lineages had only a small subpopulation capable of growing in vancomycin-free media. Many VAN-exposed lineages exhibited vancomycin dependence, which we defined as long (t_0_ > 15 h) and variable (t_0_ std. dev. > 1.5 h) lag times in the absence of vancomycin (dashed vertical and horizontal lines in **A**). (**B-C**) Example growth curves of two VAN-exposed lineages grown in the presence and absence of vancomycin (n = 24 per condition). (**D**) Population sizes in the presence and absence of vancomycin were calculated by a dilution to extinction experiment (see Materials and Methods). Shown are the relative population sizes of each VAN-exposed lineage viable in the absence of vancomycin. (**E**) Some lineages with a low fraction viability (*fv*) in the absence of vancomycin harbored a large deletion in *ddl* enabling the design of primers that targeted the deleted region. Late amplification of this region occurred for some tested lineages, indicating the presence of a small subpopulation with wild type *ddl*. The x-axis labels indicate the fraction viable in the absence of vancomycin for each lineage tested. (**F-G**) ∆Lag time (evolved—ancestor) before (**F**) and after (**G**) correcting for effective inoculum size as calculated in the dilution to extinction experiment.
(TIFF)

S6 Fig Many VAN-exposed lineages exhibited abnormal colony morphology following the removal of vancomycin (cycle 51). Images of colonies after 66 hours of growth during cycle 51 (**A**) and cycle 60 (B). (**A**) Colony morphology was manually classified as “abnormal” where indicated. (**B**) The abnormal colony morphology phenotype was no longer observed during cycle 60. Colony images were cropped and spliced from larger scans for clarity and to save room. Adjustments to brightness were applied uniformly to each strain-media-cycle group.
(TIFF)

S7 Fig Mutations in *ddl* were related to colony phenotype and the stability of vancomycin resistance. As shown in S6 Fig, many VAN-exposed lineages exhibited abnormal colony morphology. (**A**) Most *ddl* mutants exhibited abnormal colony morphology in the absence of vancomycin (two-sided Fisher’s exact test p-value = 3.2E-9). (**B**) Excluding VRSA-6 for which mutations in *ddl* and reversion to vancomycin susceptibility were rare, lineages with *ddl* mutations at cycle 60 tended to maintain resistance through cycle 60 (two-sided Fisher’s exact test p-value = 5.7E-11). (**C**) One VAN-exposed lineage of VRSA-3 underwent “pseudogene repair” between cycles 50 and 60. By cycle 50, a frameshift mutation due to a 1 base pair insertion had resulted in an early stop codon. By cycle 60, a four base pair deletion restored the frame, resulting in an amino acid deletion (Q301) and I302L substitution. Nucleotide and amino acid positions shown above the alignments refer to the ancestral sequence.
(TIFF)

S8 Fig Minimum inhibitory concentration of vancomycin, sulfamethoxazole-trimethoprim, oxacillin, and linezolid after 50 cycles of propagation. Horizontal dashed lines indicate the clinical breakpoint for each drug as defined in CLSI M100. Black horizontal lines indicate the MIC of ancestral strains as measured by broth microdilution in BHI and TSB. Black points (x) on the vertical dashed lines indicate the MIC of ancestral strains as measured by broth microdilution in cation-adjusted MHB. All MICs are given in units of μg/ml. (**A**) Vancomycin MIC of VAN-exposed lineages remained unchanged. (**B**) Vancomycin MIC decreased sharply in many VAN-unexposed lineages with the exception of most VRSA-6 lineages. (**C-D**) Most BHI-propagated lineages were resistant to sulfamethoxazole-trimethoprim (SXT), while TSB-propagated lineages were susceptible. Ancestral strains were SXT resistant as measured in BHI/TSB, but not MHB, with the exception of VRSA-6. Y-axis labels indicate the concentration of sulfamethoxazole and trimethoprim (e.g., 0.5/9 refers to 0.5 μg/ml sulfamethoxazole and 9 μg/ml trimethoprim). (**E-F**) Oxacillin MIC decreased in most lineages, but the magnitude of change was different between VAN-exposed and VAN-unexposed lineages. VAN-exposed lineages generally had lower oxacillin MICs than VAN-unexposed lineages. (**G-H**) Linezolid MIC remained largely unchanged in VAN-exposed and VAN-unexposed lineages.
(TIFF)

S9 Fig Oxacillin MICs of cycle 50 and cycle 60 VAN-exposed lineages. Horizontal dashed lines indicate the clinical breakpoint for each drug as defined in CLSI M100. Black horizontal lines indicate the MIC of ancestral strains as measured by broth microdilution in BHI and TSB. Black points (x) on the vertical dashed lines indicate the MIC of ancestral strains as measured by broth microdilution in cation-adjusted Mueller-Hinton Broth. All MICs are given in units of μg/ml. Oxacillin MICs increased slightly between (**A**) cycle 50 and (**B**) cycle 60 (two-sided Wilcoxon signed-rank test p-value = 0.023).
(TIFF)

S1 Table D-alanine:D-alanine ligase (*ddl*) mutations in VAN-exposed lineages and clinical VRSA isolates. Descriptions of the *ddl* mutations observed in VAN-exposed lineages generated in this study and in clinical VRSA isolates for which sequencing data is available (VRSA-1 through VRSA-11). Of the 44 lineages with a *ddl* mutation at cycle 50, 33 retained the mutation at cycle 60. Sequencing data was not available for cycle 60 VRSA-6k_BHI, which had a *ddl* mutation at cycle 50. Notably, VRSA-3c_BHI harbored a 4 base deletion (relative to cycle 50) that restored the frame of *ddl* and resulted in the deletion of Q301 and the substitution I302L relative to the wild type sequence. Additionally, VRSA-10d_BHI harbored the same nonsynonymous mutation as ancestral VRSA-6 (N308K). Nonsynonymous and nonsense mutations indicate the affected amino acid position, while insertion, deletion, and intergenic mutations indicate the affected nucleotide(s) position(s). Base 1 is the ‘A’ in start codon ‘ATG’ (‘587_598del2’ indicates that 12 nucleotides at positions 587 through 598 were deleted, ‘-70A>G’ indicates that the 70th nucleotide upstream of the start base was substituted, etc.).
(TIFF)

## References

[1] Horn DL, Zabriskie JB, Austrian R, Cleary PP, Ferretti JJ, Fischetti VA, et al. Why Have Group A Streptococci Remained Susceptible to Penicillin? Report on a Symposium. Clinical Infectious Diseases. 1998;26:1341–5. doi: 10.1086/516375 9636860

[2] Beckley AM, Wright ES. Identification of antibiotic pairs that evade concurrent resistance via a retrospective analysis of antimicrobial susceptibility test results. The Lancet Microbe. 2021;2(10):e545–e54. doi: 10.1016/s2666-5247(21)00118-x 34632433 PMC8496867

[3] Cong Y, Yang S, Rao X. Vancomycin resistant *Staphylococcus aureus* infections: A review of case updating and clinical features. J Adv Res. 2020;21:169–76. 32071785 10.1016/j.jare.2019.10.005 PMC7015472

[4] Bjorkman J, Andersson DI. The cost of antibiotic resistance from a bacterial perspective. Drug Resist Updat. 2000;3(4):237–45. doi: 10.1054/drup.2000.0147 11498391

[5] Bell G, MacLean C. The Search for ’Evolution-Proof’ Antibiotics. Trends Microbiol. 2018;26(6):471–83. doi: 10.1016/j.tim.2017.11.005 29191398

[6] Allen RC, Popat R, Diggle SP, Brown SP. Targeting virulence: can we make evolution-proof drugs? Nat Rev Microbiol. 2014;12(4):300–8. doi: 10.1038/nrmicro3232 24625893

[7] Zhang Y, Chowdhury S, Rodrigues JV, Shakhnovich E. Development of antibacterial compounds that constrain evolutionary pathways to resistance. Elife. 2021;10(e64518). doi: 10.7554/eLife.64518 34279221 PMC8331180

[8] Chait R, Vetsigian K, Kishony R. What counters antibiotic resistance in nature? Nat Chem Biol. 2011;8(1):2–5. doi: 10.1038/nchembio.745 22173342

[9] Waglechner N, Wright GD. Antibiotic resistance: it’s bad, but why isn’t it worse? BMC Biol. 2017;15(1):84. doi: 10.1186/s12915-017-0423-1 28915805 PMC5603022

[10] Kirst H, Thompson D, Nicas T. Historical Yearly Usage of Vancomycin. Antimicrob Agents and Chemotherapy. 1998;42(5):1303–4. doi: 10.1128/AAC.42.5.1303 9593175 PMC105816

[11] Centers for Disease Control and Prevention. Active Bacterial Core Surveillance Report, Emerging Infections Program Network, Methicillin Resistant *Staphylococcus aureus*, 2014. 2016.

[12] Centers for Disease Control and Prevention. Clinician Brief: Clinical Laboratories’ and Infection Preventionists’ Roles in the Search for and Containment of Vancomycin-Resistant *Staphylococcus aureus* [Web Page]. 2022

[13] Vehreschild M, Haverkamp M, Biehl LM, Lemmen S, Fatkenheuer G. Vancomycin-resistant enterococci (VRE): a reason to isolate? Infection. 2019;47(1):7–11. doi: 10.1007/s15010-018-1202-9 30178076

[14] Gales AC, Sader HS, Andrade SS, Lutz L, Machado A, Barth AL. Emergence of linezolid-resistant *Staphylococcus aureus* during treatment of pulmonary infection in a patient with cystic fibrosis. Int J Antimicrob Agents. 2006;27(4):300–2. doi: 10.1016/j.ijantimicag.2005.11.008 16527459

[15] Besier S, Ludwig A, Zander J, Brade V, Wichelhaus TA. Linezolid resistance in *Staphylococcus aureus*: gene dosage effect, stability, fitness costs, and cross-resistances. Antimicrob Agents Chemother. 2008;52(4):1570–2. 18212098 10.1128/AAC.01098-07 PMC2292563

[16] Hayden MK, Rezai K, Hayes RA, Lolans K, Quinn JP, Weinstein RA. Development of Daptomycin resistance in vivo in methicillin-resistant *Staphylococcus aureus*. J Clin Microbiol. 2005;43(10):5285–7. 16207998 10.1128/JCM.43.10.5285-5287.2005 PMC1248493

[17] Friedman L, Alder JD, Silverman JA. Genetic changes that correlate with reduced susceptibility to daptomycin in *Staphylococcus aureus*. Antimicrob Agents Chemother. 2006;50(6):2137–45. 16723576 10.1128/AAC.00039-06 PMC1479123

[18] Hiramatsu K, Hanaki H, Ino T, Yatuba K, Oguri T, Tenover FC. Methicillin-Resistant *Staphylococcus aureus* Clinical Strain with Reduced Vancomycin Susceptibility. Journal of Antimicrobial Chemotherapy. 1997;40:135–46. 9249217 10.1093/jac/40.1.135

[19] Cui L, Ma X, Sato K, Okuma K, Tenover FC, Mamizuka EM, et al. Cell wall thickening is a common feature of vancomycin resistance in *Staphylococcus aureus*. J Clin Microbiol. 2003;41(1):5–14. 12517819 10.1128/JCM.41.1.5-14.2003 PMC149586

[20] Flannagan SE, Chow JW, Donabedian SM, Brown WJ, Perri MB, Zervos MJ, et al. Plasmid content of a vancomycin-resistant *Enterococcus faecalis* isolate from a patient also colonized by Staphylococcus aureus with a VanA phenotype. Antimicrob Agents Chemother. 2003;47(12):3954–9. 14638508 10.1128/AAC.47.12.3954-3959.2003 PMC296223

[21] Weigel LM, Clewell DB, Gill SR, Clark NC, McDougal LK, Flannagan SE, et al. Genetic Analysis of a High-Level Vancomycin-Resistant Isolate of *Staphylococcus aureus*. Science. 2003;302:1569–71. 14645850 10.1126/science.1090956

[22] Chang S, Sievert D, Hageman J, Boulton M, Tenover F, Downes F, et al. Infection with Vancomycin-Resistant *Staphylococcus aureus* Containing the *vanA* Resistance Gene. N Engl J Med. 2003;384(14):1342–7. 10.1056/NEJMoa025025 12672861

[23] Whitener CJ, Park SY, Browne FA, Parent LJ, Julian K, Bozdogan Bl et al. Vancomycin-Resistant *Staphylococcus aureus* in the Absence of Vancomycin Exposure. Clinical Infectious Diseases. 2004;38:1049–55. 15095205 10.1086/382357

[24] Weigel LM, Donlan RM, Shin DH, Jensen B, Clark NC, McDougal LK, et al. High-level vancomycin-resistant *Staphylococcus aureus* isolates associated with a polymicrobial biofilm. Antimicrob Agents Chemother. 2007;51(1):231–8. 17074796 10.1128/AAC.00576-06 PMC1797660

[25] Finks J, Wells E, Dyke TL, Husain N, Plizga L, Heddurshetti R, et al. Vancomycin-resistant *Staphylococcus aureus*, Michigan, USA, 2007. Emerg Infect Dis. 2009;15(6):943–5. 19523298 10.3201/eid1506.081312 PMC2727339

[26] Kos VN, Desjardins CA, Griggs A, Cerqueira G, Van Tonder A, Holden MT, et al. Comparative genomics of vancomycin-resistant *Staphylococcus aureus* strains and their positions within the clade most commonly associated with Methicillin-resistant *S*. *aureus* hospital-acquired infection in the United States. mBio. 2012;3(3). 10.1128/mBio.00112-12 PMC3372964 22617140

[27] Limbago BM, Kallen AJ, Zhu W, Eggers P, McDougal LK, Albrecht VS. Report of the 13th vancomycin-resistant *Staphylococcus aureus* isolate from the United States. J Clin Microbiol. 2014;52(3):998–1002. 24371243 10.1128/JCM.02187-13 PMC3957794

[28] Rossi F, Diaz L, Wollam A, Panesso D, Zhou Y, Rincon S, et al. Transferable vancomycin resistance in a community-associated MRSA lineage. N Engl J Med. 2014;370(16):1524–31. doi: 10.1056/NEJMoa1303359 24738669 PMC4112484

[29] Walters MS, Eggers P, Albrecht V, Travis T, Lonsway D, Hovan G, et al. Vancomycin-Resistant *Staphylococcus aureus*—Delaware, 2015. Morbidity and Mortality Weekly Report. 2015;64(37):1056–7. 26402026 10.15585/mmwr.mm6437a6

[30] Koyama N, Inokoshi J, Tomoda H. Anti-infectious agents against MRSA. Molecules. 2012;18(1):204–24. doi: 10.3390/molecules18010204 23262449 PMC6269750

[31] Reynolds PE. Structure, Biochemistry and Mechanism of Action of Glycopeptide Antibiotics. Eur J Clin Microbiol Infect Dis. 1989;8(11):943–50. doi: 10.1007/BF01967563 2532132

[32] Stogios PJ, Savchenko A. Molecular mechanisms of vancomycin resistance. Protein Sci. 2020;29(3):654–69. doi: 10.1002/pro.3819 31899563 PMC7020976

[33] Bugg TDH, Wright GD, Dutka-Malen S, Arthur M, Courvalin P, Walsh CT. Molecular Basis for Vancomycin Resistance in *Enterococcus faecium* BM4147: Biosynthesis of a Depsipeptide Peptidoglycan Precursor by Vancomycin Resistance Proteins VanH and VanA. Biochemistry. 1991;30:10408–15. 1931965 10.1021/bi00107a007

[34] Sievert DM, Rudrik JT, Patel JB, McDonald LC, Wilkins MJ, Hageman JC. Vancomycin-resistant *Staphylococcus aureus* in the United States, 2002–2006. Clin Infect Dis. 2008;46(5):668–74. 18257700 10.1086/527392

[35] Perichon B, Courvalin P. Heterologous expression of the enterococcal vanA operon in methicillin-resistant *Staphylococcus aureus*. Antimicrob Agents Chemother. 2004;48(11):4281–5. 15504853 10.1128/AAC.48.11.4281-4285.2004 PMC525442

[36] Foucault ML, Courvalin P, Grillot-Courvalin C. Fitness cost of VanA-type vancomycin resistance in methicillin-resistant *Staphylococcus aureus*. Antimicrob Agents Chemother. 2009;53(6):2354–9. 19332680 10.1128/AAC.01702-08 PMC2687198

[37] Perichon B, Courvalin P. *Staphylococcus aureus* VRSA-11B is a constitutive vancomycin-resistant mutant of vancomycin-dependent VRSA-11A. Antimicrob Agents Chemother. 2012;56(9):4693–6. 22710116 10.1128/AAC.00454-12 PMC3421854

[38] Perichon B, Courvalin P. Synergism between beta-lactams and glycopeptides against VanA-type methicillin-resistant *Staphylococcus aureus* and heterologous expression of the vanA operon. Antimicrob Agents Chemother. 2006;50(11):3622–30. 16954318 10.1128/AAC.00410-06 PMC1635195

[39] Meziane-Cherif D, Saul FA, Moubareck C, Weber P, Haouz A, Courvalin P, et al. Molecular basis of vancomycin dependence in VanA-type *Staphylococcus aureus* VRSA-9. J Bacteriol. 2010;192(20):5465–71. 20729361 10.1128/JB.00613-10 PMC2950510

[40] Moubareck C, Meziane-Cherif D, Courvalin P, Perichon B. VanA-type *Staphylococcus aureus* strain VRSA-7 is partially dependent on vancomycin for growth. Antimicrob Agents Chemother. 2009;53(9):3657–63. 19528271 10.1128/AAC.00338-09 PMC2737872

[41] Rosato A, Pierre J, Billot-Klein D, Buu-Hoi A, Gutmann L. Inducible and Constitutive Expression of Resistance to Glycopeptides and Vancomycin Dependence in Glycopeptide-Resistant *Enterococcus avium*. Antimicrob Agents and Chemotherapy. 1995;39(4):830–3. doi: 10.1128/AAC.39.4.830 7785979 PMC162637

[42] Kirkpatrick BD, Harrington SM, Smith D, Marcellus D, Miller C, Dick J, et al. An Outbreak of Vancomycin-Dependent *Enterococcus faecium* in a Bone Marrow Transplant Unit. Clinical Infectious Diseases. 1999;29:1268–73. 10524974 10.1086/313456

[43] Fraimow HS, Jungkind DL, Lander DW, Delso DR, Dean JL. Urinary Tract Infection with an *Enterococcus faecalis* Isolate that Requires Vancomycin for Growth. Ann Intern Med. 1994;121:22–6. 8198343 10.7326/0003-4819-121-1-199407010-00004

[44] Schrag SJ, Perrot V, Levin BR. Adaptation to the fitness costs of antibiotic resistance in *Escherichia coli*. Proc R Soc Lond B. 1997;264:1287–91. 10.1098/rspb.1997.0178 PMC1688596 9332013

[45] Andersson DI, Levin BR. The biological cost of antibiotic resistance. Current Opinion in Microbiology. 1999;2:489–93. doi: 10.1016/s1369-5274(99)00005-3 10508723

[46] Levin BR, Perrot V, Walker N. Compensatory Mutations, Antibiotic Resistance and the Population Genetics of Adaptive Evolution in Bacteria. Genetics. 2000;154:985–97. doi: 10.1093/genetics/154.3.985 10757748 PMC1460977

[47] Maisnier-Patin S, Berg OG, Liljas L, Andersson DI. Compensatory adaptation to the deleterious effect of antibiotic resistance in *Salmonella typhimurium*. Mol Microbiol. 2002;46(2):355–66. 12406214 10.1046/j.1365-2958.2002.03173.x

[48] Fait A, Andersson DI, Ingmer H. Evolutionary history of *Staphylococcus aureus* influences antibiotic resistance evolution. Curr Biol. 2023;33(16):3389–97 e5. 37494936 10.1016/j.cub.2023.06.082

[49] Touati A, Bellil Z, Barache D, Mairi A. Fitness Cost of Antibiotic Resistance in *Staphylococcus aureus*: A Systematic Review. Microb Drug Resist. 2021;27(9):1218–31. 33417813 10.1089/mdr.2020.0426

[50] Johnsen PJ, Simonsen GS, Olsvik Ø, Midtvedt T, Sundsfjord A. Stability, Persistence, and Evolution of Plasmid-Encoded VanA Glycopeptide Resistance in Enterococci in the Absence of Antibiotic Selection *In Vitro* and in Gnotobiotic Mice. Microbial Drug Resistance. 2002;8(3):161–70. 12363004 10.1089/107662902760326869

[51] Lai KK, Fontecchio SA, Kelley AL, Melvin ZS, Baker S. The Epidemiology of Fecal Carriage of Vancomycin-Resistant Enterococci. Infection Control and Hospital Epidemiology. 1997;18(11):762–5. 9397370

[52] Besier S, Ludwig A, Brade V, Wichelhaus TA. Compensatory adaptation to the loss of biological fitness associated with acquisition of fusidic acid resistance in *Staphylococcus aureus*. Antimicrob Agents Chemother. 2005;49(4):1426–31. 15793122 10.1128/AAC.49.4.1426-1431.2005 PMC1068613

[53] Nagaev I, Bjorkman J, Andersson DI, Hughes D. Biological cost and compensatory evolution in fusidic acid-resistant *Staphylococcus aureus*. Mol Microbiol. 2001;40(2):433–9. 11309125 10.1046/j.1365-2958.2001.02389.x

[54] Andersson DI. Persistence of antibiotic resistant bacteria. Curr Opin Microbiol. 2003;6(5):452–6. doi: 10.1016/j.mib.2003.09.001 14572536

[55] Martin JH, Norris R, Barras M, Roberts J, Morris R, Doogue M, et al. Therapeutic Monitoring of Vancomycin in Adult Patients: A Consensus Review of the American Society of Health-System Pharmacists, the Infectious Diseases Society of America, and the Society of Infectious Diseases Pharmacists. Clin Biochem Rev. 2010;31:21–4. 20179794 PMC2826264

[56] Casadewall B, Courvalin P. Characterization of the *vanD* Glycopeptide Resistance Gene Cluster from *Enterococcus faecium* BM4339. Journal of Bacteriology. 1999;181(12):3644–8. 10368136 10.1128/jb.181.12.3644-3648.1999 PMC93839

[57] Anand A, Olson CA, Yang L, Sastry AV, Catoiu E, Choudhary KS, et al. Pseudogene repair driven by selection pressure applied in experimental evolution. Nat Microbiol. 2019;4(3):386–9. doi: 10.1038/s41564-018-0340-2 30692668

[58] Severin A, Tabei K, Tenover F, Chung M, Clarke N, Tomasz A. High level oxacillin and vancomycin resistance and altered cell wall composition in *Staphylococcus aureus* carrying the staphylococcal *mecA* and the enterococcal *vanA* gene complex. J Biol Chem. 2004;279(5):3398–407. 14613936 10.1074/jbc.M309593200

[59] Arthur M, Molinas C, Courvalin P. The VanS-VanR Two-Component Regulatory System Controls Synthesis of Depsipeptide Peptidoglycan Precursors in *Enterococcus faecium* BM4147. Journal of Bacteriology. 1992;174(8):2582–91. 1556077 10.1128/jb.174.8.2582-2591.1992 PMC205897

[60] Harkins CP, Pichon B, Doumith M, Parkhill J, Westh H, Tomasz A, et al. Methicillin-resistant *Staphylococcus aureus* emerged long before the introduction of methicillin into clinical practice. Genome Biol. 2017;18(1):130. 28724393 10.1186/s13059-017-1252-9 PMC5517843

[61] Habets MG, Czaran T, Hoekstra RF, de Visser JA. Spatial structure inhibits the rate of invasion of beneficial mutations in asexual populations. Proc Biol Sci. 2007;274(1622):2139–43. doi: 10.1098/rspb.2007.0529 17609190 PMC2706196

[62] Fusco D, Gralka M, Kayser J, Anderson A, Hallatschek O. Excess of mutational jackpot events in expanding populations revealed by spatial Luria-Delbruck experiments. Nat Commun. 2016;7:12760. 27694797 10.1038/ncomms12760 PMC5059437

[63] Lenski RE. Quantifying Fitness and Gene Stability in Microorganisms. Assessing Ecological Risks of Biotechnology. 1991:173–92. doi: 10.1016/b978-0-409-90199-3.50015-2 2009380

[64] Perichon B, Courvalin P. VanA-type vancomycin-resistant *Staphylococcus aureus*. Antimicrob Agents Chemother. 2009;53(11):4580–7. 19506057 10.1128/AAC.00346-09 PMC2772335

[65] Hall AR, Iles JC, MacLean RC. The fitness cost of rifampicin resistance in *Pseudomonas aeruginosa* depends on demand for RNA polymerase. Genetics. 2011;187(3):817–22. 21220359 10.1534/genetics.110.124628 PMC3063675

[66] Tedim AP, Lanza VF, Rodriguez CM, Freitas AR, Novais C, Peixe L, et al. Fitness cost of vancomycin-resistant *Enterococcus faecium* plasmids associated with hospital infection outbreaks. J Antimicrob Chemother. 2021;76(11):2757–64. 34450635 10.1093/jac/dkab249

[67] Okano A, Nakayama A, Wu K, Lindsey EA, Schammel AW, Feng Y, et al. Total syntheses and initial evaluation of [Ψ[C (= S)NH]Tpg(4)]vancomycin, [Ψ[C (= NH)NH]Tpg(4)]vancomycin, [Ψ[CH(2)NH]Tpg(4)]vancomycin, and their (4-chlorobiphenyl)methyl derivatives: synergistic binding pocket and peripheral modifications for the glycopeptide antibiotics. J Am Chem Soc. 2015;137(10):3693–704. 25750995 10.1021/jacs.5b01008 PMC4376669

[68] Koulenti D, Xu E, Mok IYS, Song A, Karageorgopoulos DE, Armaganidis A, et al. Novel Antibiotics for Multidrug-Resistant Gram-Positive Microorganisms. Microorganisms. 2019;7(8). 10.3390/microorganisms7080270 PMC6723731 31426596

[69] Magana M, Pushpanathan M, Santos AL, Leanse L, Fernandez M, Ioannidis A, et al. The value of antimicrobial peptides in the age of resistance. Lancet Infect Dis. 2020;20(9):e216–e30. doi: 10.1016/S1473-3099(20)30327-3 32653070

[70] Suh GA, Lodise TP, Tamm PD, Knisely JM, Alexander J, Aslam S, et al. Considerations for the Use of Phage Therapy in Clinical Practice. Antimicrob Agents and Chemotherapy. 2022;66(3). doi: 10.1128/AAC.02071-21 35041506 PMC8923208

[71] Hatfull GF, Dedrick RM, Schooley RT. Phage Therapy for Antibiotic-Resistant Bacterial Infections. Annu Rev Med. 2022;73:197–211. doi: 10.1146/annurev-med-080219-122208 34428079

[72] Theuretzbacher U, Outterson K, Engel A, Karlen A. The global preclinical antibacterial pipeline. Nat Rev Microbiol. 2020;18(5):275–85. doi: 10.1038/s41579-019-0288-0 31745331 PMC7223541

[73] Schneider CA, Rasband WS, Eliceiri KW. NIH Image to ImageJ: 25 years of image analysis. Nat Methods. 2012;9(7):671–5. doi: 10.1038/nmeth.2089 22930834 PMC5554542

[74] R Core Team. R: A language and environment for statistical computing. 2022.

[75] Therneau TM, Grambsch PM. Modeling Survival Data: Extending the Cox Model. Springer. 2000.

[76] Jones A, Stanley D, Ferguson S, Schwessinger B, Borevitz J, Warthmann N. Cost-conscious generation of multiplexed short-read DNA libraries for whole-genome sequencing. PLoS One. 2023;18(1):e0280004. doi: 10.1371/journal.pone.0280004 36706059 PMC9882895

[77] Bankevich A, Nurk S, Antipov D, Gurevich AA, Dvorkin M, Kulikov AS, et al. SPAdes: a new genome assembly algorithm and its applications to single-cell sequencing. J Comput Biol. 2012;19(5):455–77. doi: 10.1089/cmb.2012.0021 22506599 PMC3342519

[78] Deatherage DE, Barrick JE. Identification of mutations in laboratory-evolved microbes from next-generation sequencing data using breseq. Methods Mol Biol. 2014;1151:165–88. doi: 10.1007/978-1-4939-0554-6_12 24838886 PMC4239701

