# Supplementary figures and images for: Vancomycin-resistant Staphylococcus aureus (VRSA) can overcome the cost of antibiotic resistance and may threaten vancomycin’s clinical durability
Source: PLoS Pathog. 2024 Aug 29;20(8):e1012422. doi: 10.1371/journal.ppat.1012422 (PMC11361437; doi:10.1371/journal.ppat.1012422)

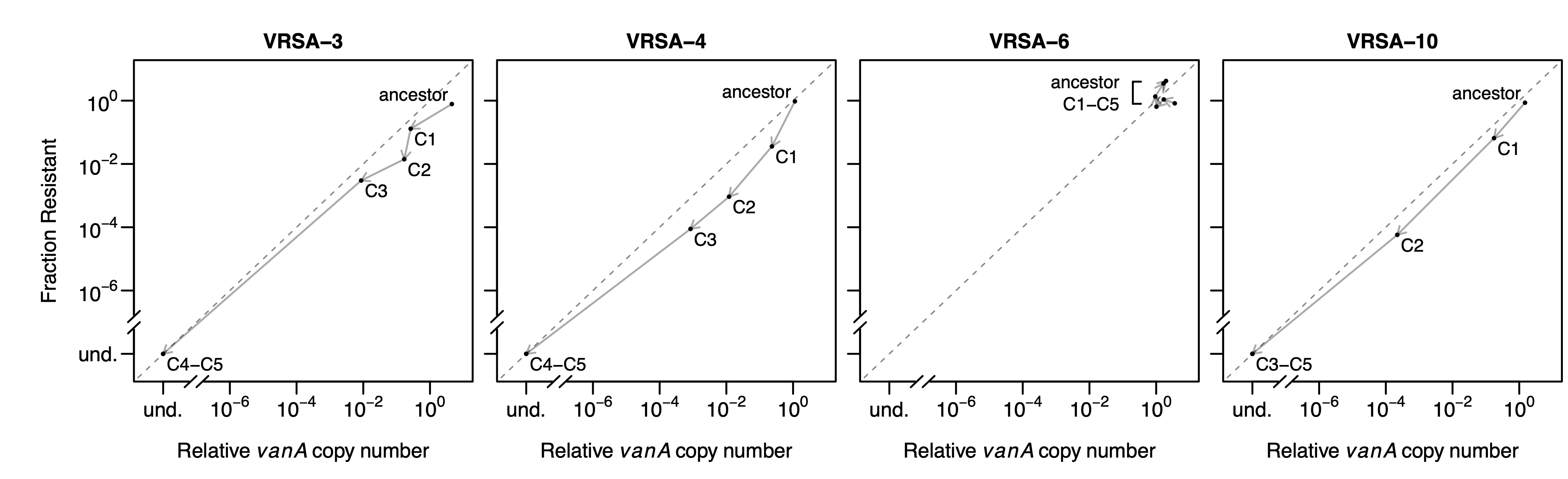

Supplement: S1 Fig — One sample of each strain was propagated on BHI agar (0 μg/ml vancomycin) for 5 propagation cycles. As described in Materials and Methods, the perimeter of the colonies was harvested and we measured the fraction of cells that retained resistance and the relative copy number of the vanA gene (relative to aroE, see Materials and Methods). ‘C1’, ‘C2’, etc. refers to propagation cycle 1, 2, etc. (TIFF) [file ppat.1012422.s001.tiff]

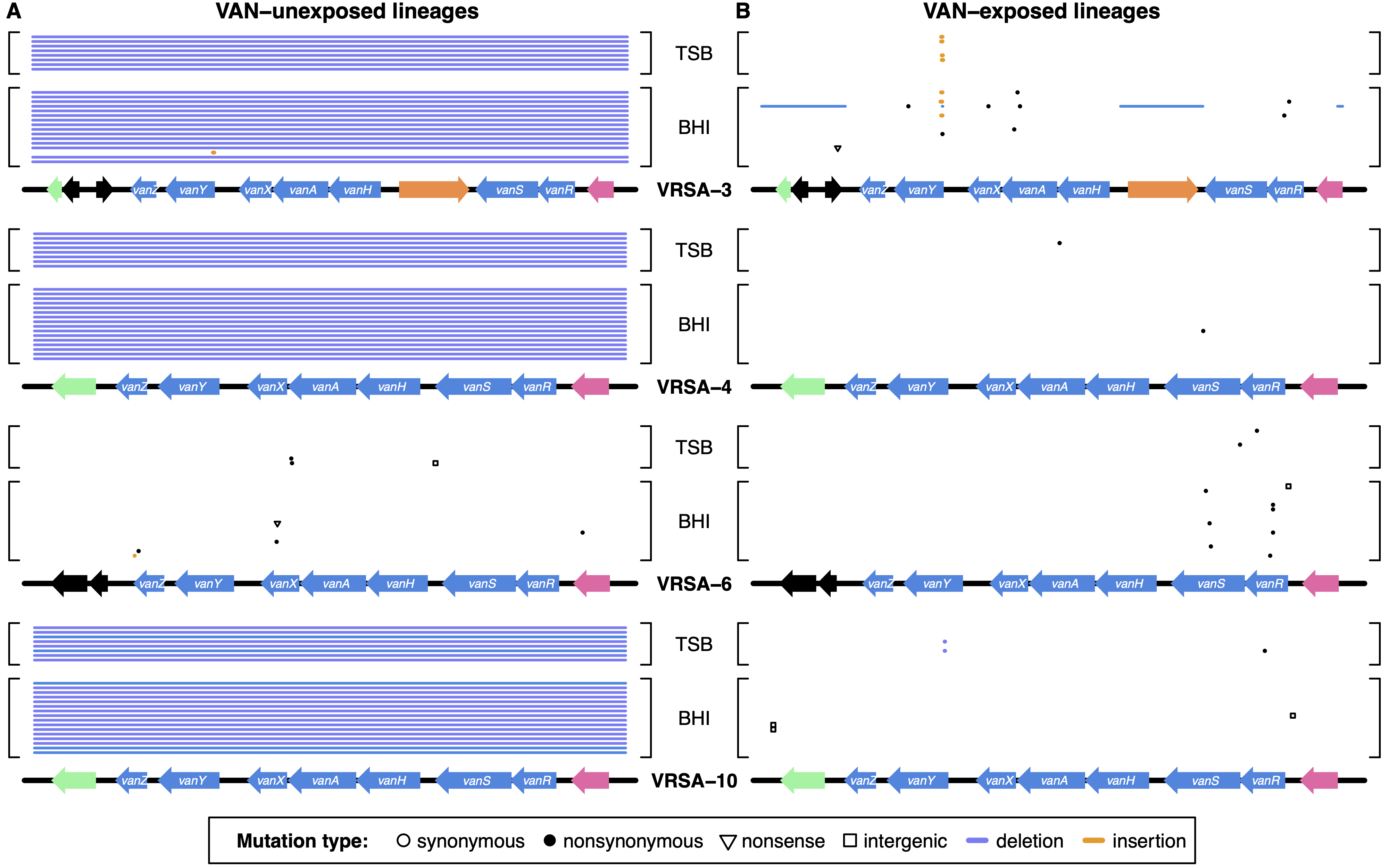

Supplement: S2 Fig — Mutations in the vanA operon and flanking gene(s) in cycle 50 VAN-unexposed (A) and VAN-exposed (B) lineages. (A) Most Cycle 50 VAN-unexposed lineages of VRSA-3, -4, and -10 show loss of the entire contig on which the vanA operon resides, consistent with their loss of resistance. A VRSA-3 lineage maintained the vanA operon, but had a 12 bp insertion in vanY and was susceptible to vancomycin. VRSA-6 maintained resistance in all but one BHI-propagated lineage which harbored a nonsense mutation in vanX. Some VRSA-6 lineages had mutations in some vanA operon genes. (B) Numerous and varied mutations were seen in cycle 50 VAN-exposed lineages throughout the vanA operon in some strains. Several genes appeared to have mutations across multiple lineages, including vanR and vanS in VRSA-6. (TIFF) [file ppat.1012422.s002.tiff]

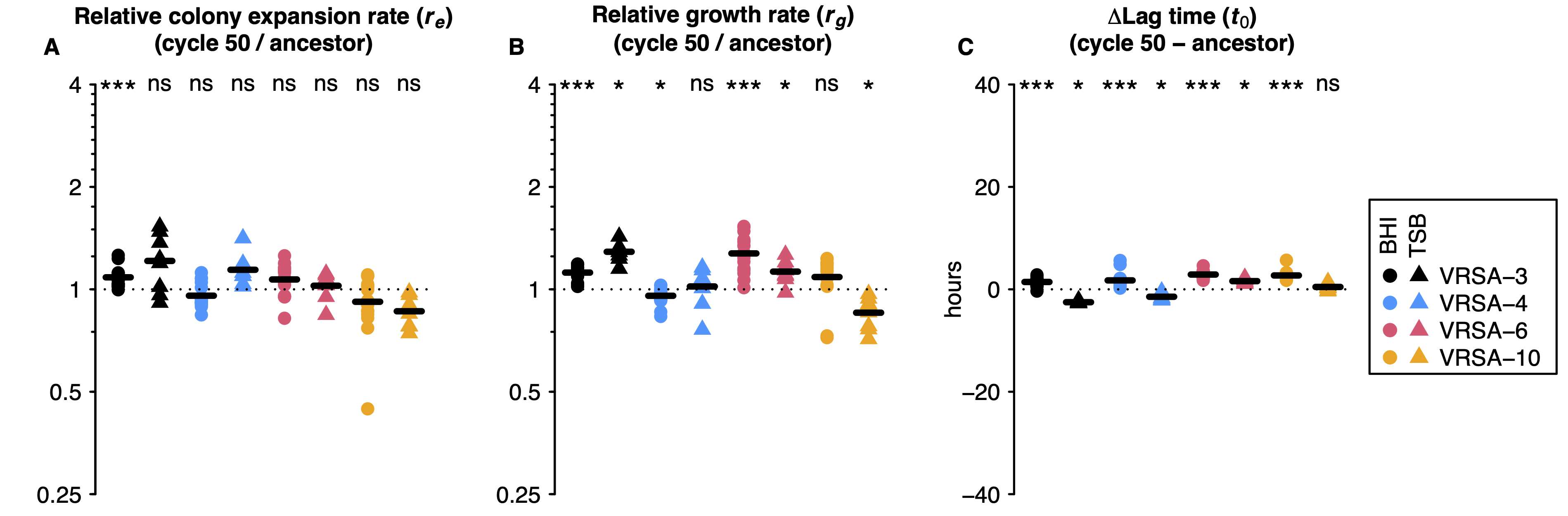

Supplement: S3 Fig — Fitness changes in VAN-unexposed lineages did not follow a trend in either direction in colony expansion rate (A), growth rate (B), and lag time (C). Colony expansion rate was measured once per evolved lineage, while growth rate and lag time were taken from the mean of 24 technical replicates (see Materials and Methods). Each group was compared to the mean of the corresponding ancestral strain by a two-sided Wilcoxon signed-rank test. Asterisks denote statistical significance after Holm-Bonferroni multiple testing correction on a panel-by-panel basis (*** = p-adj. < 0.001, ** = p-adj. < 0.01, * = p-adj. < 0.05, ns = p-adj. ≥ 0.05). (TIFF) [file ppat.1012422.s003.tiff]

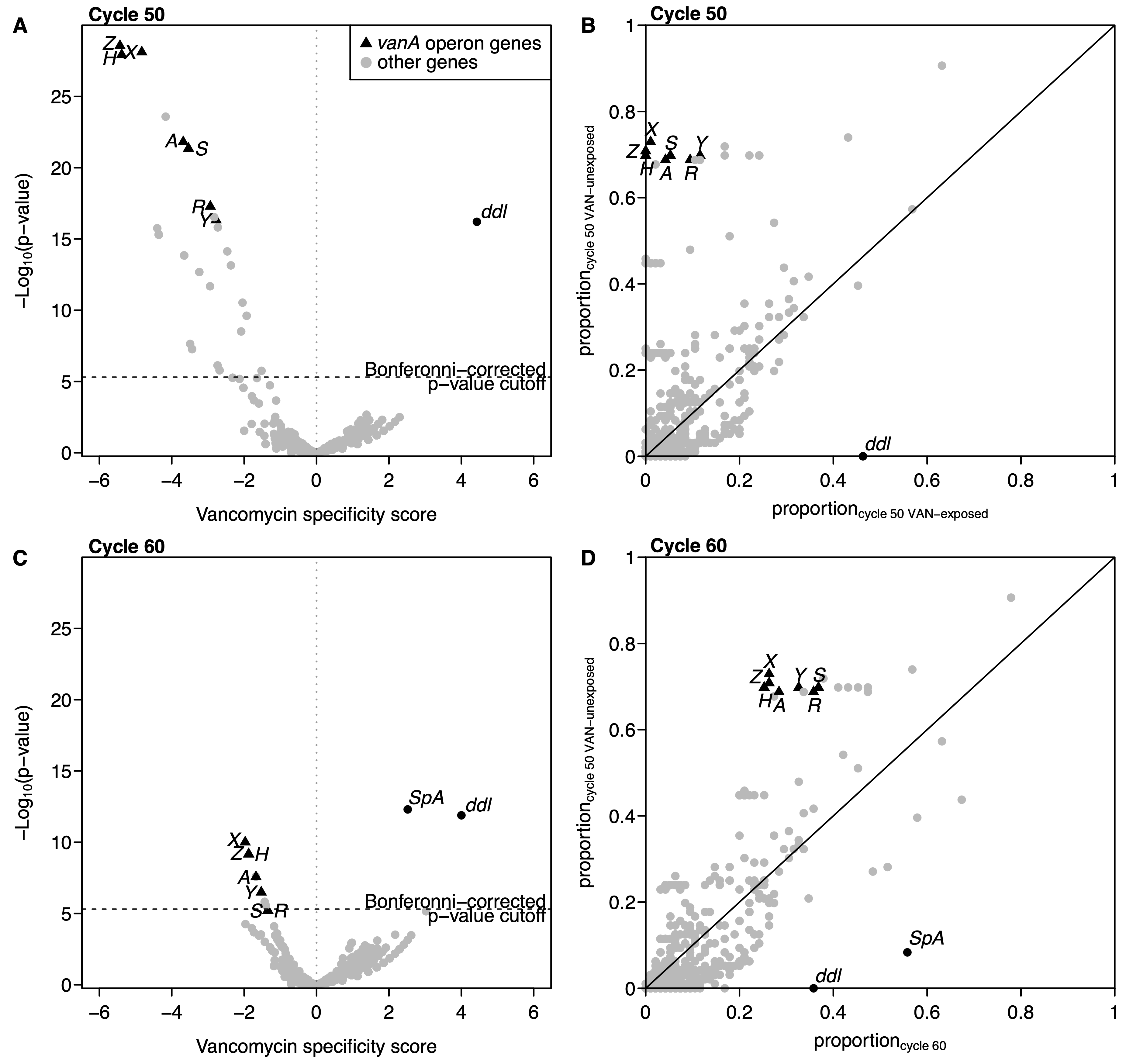

Supplement: S4 Fig — Genes were ranked according to their vancomycin specificity score (see Materials and Methods) using mutation data of evolved lineages at cycle 50 (A-B) and cycle 60 (C-D). (A) Volcano plot showing the vancomycin specificity score and its statistical significance for each gene at cycle 50. (B) A scatter plot showing the proportion of each group that harbored at least one mutation in each gene. Genes in and around the vanA operon appeared in the upper left corner (i.e., they occurred in VAN-unexposed lineages more often) due to segregational loss of the vanA plasmid and ddl in the lower right. (C) Relative to cycle 50, genes of the vanA operon have shifted down and to the right due to plasmid loss in several lineages that reverted to susceptibility or due to the acquisition of point mutations in lineages that retained resistance through cycle 60. Finally, ddl has shifted left and down relative to cycle 50 due to several lineages that apparently “reverted” to wild type ddl by cycle 60. (D) Relative to cycle 50, genes of the vanA operon have shifted to the right and ddl to the left for the same reasons as described previously. SpA refers to the gene staphylococcal protein A. P-values were calculated using Fisher’s exact test (two-sided fisher.test) for a difference in the proportions of each group that had at least one mutation in a gene. The p-value cutoff was calculated using the Bonferroni procedure considering the number of possible genes that were tested (n = 2,046). The single italicized letters refer to the genes of the vanA operon (i.e., vanRSHAXYZ). (TIFF) [file ppat.1012422.s004.tiff]

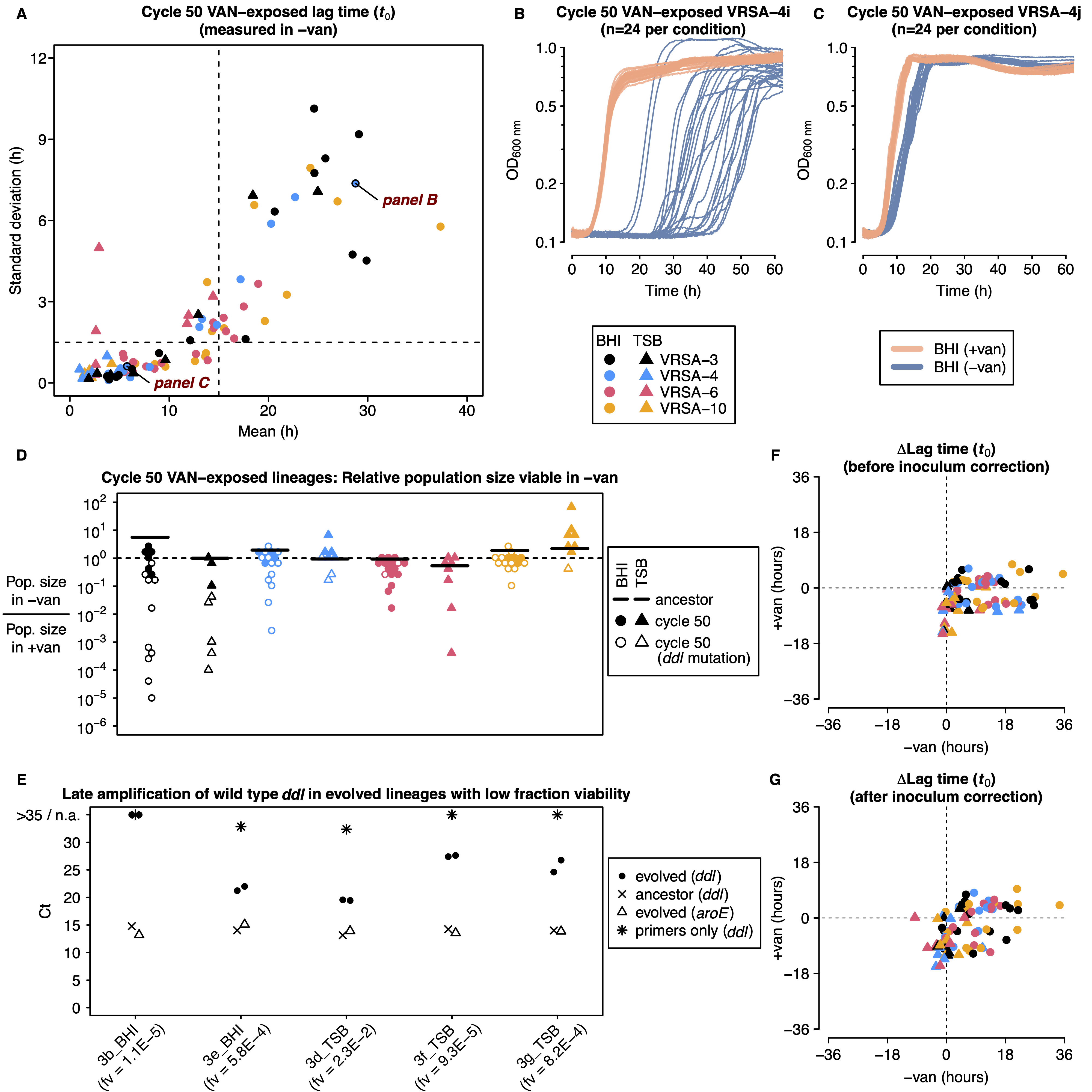

Supplement: S5 Fig — Many VAN-exposed lineages exhibited vancomycin dependence, which we defined as long (t0 > 15 h) and variable (t0 std. dev. > 1.5 h) lag times in the absence of vancomycin (dashed vertical and horizontal lines in A). (B-C) Example growth curves of two VAN-exposed lineages grown in the presence and absence of vancomycin (n = 24 per condition). (D) Population sizes in the presence and absence of vancomycin were calculated by a dilution to extinction experiment (see Materials and Methods). Shown are the relative population sizes of each VAN-exposed lineage viable in the absence of vancomycin. (E) Some lineages with a low fraction viability (fv) in the absence of vancomycin harbored a large deletion in ddl enabling the design of primers that targeted the deleted region. Late amplification of this region occurred for some tested lineages, indicating the presence of a small subpopulation with wild type ddl. The x-axis labels indicate the fraction viable in the absence of vancomycin for each lineage tested. (F-G) ∆Lag time (evolved—ancestor) before (F) and after (G) correcting for effective inoculum size as calculated in the dilution to extinction experiment. (TIFF) [file ppat.1012422.s005.tiff]

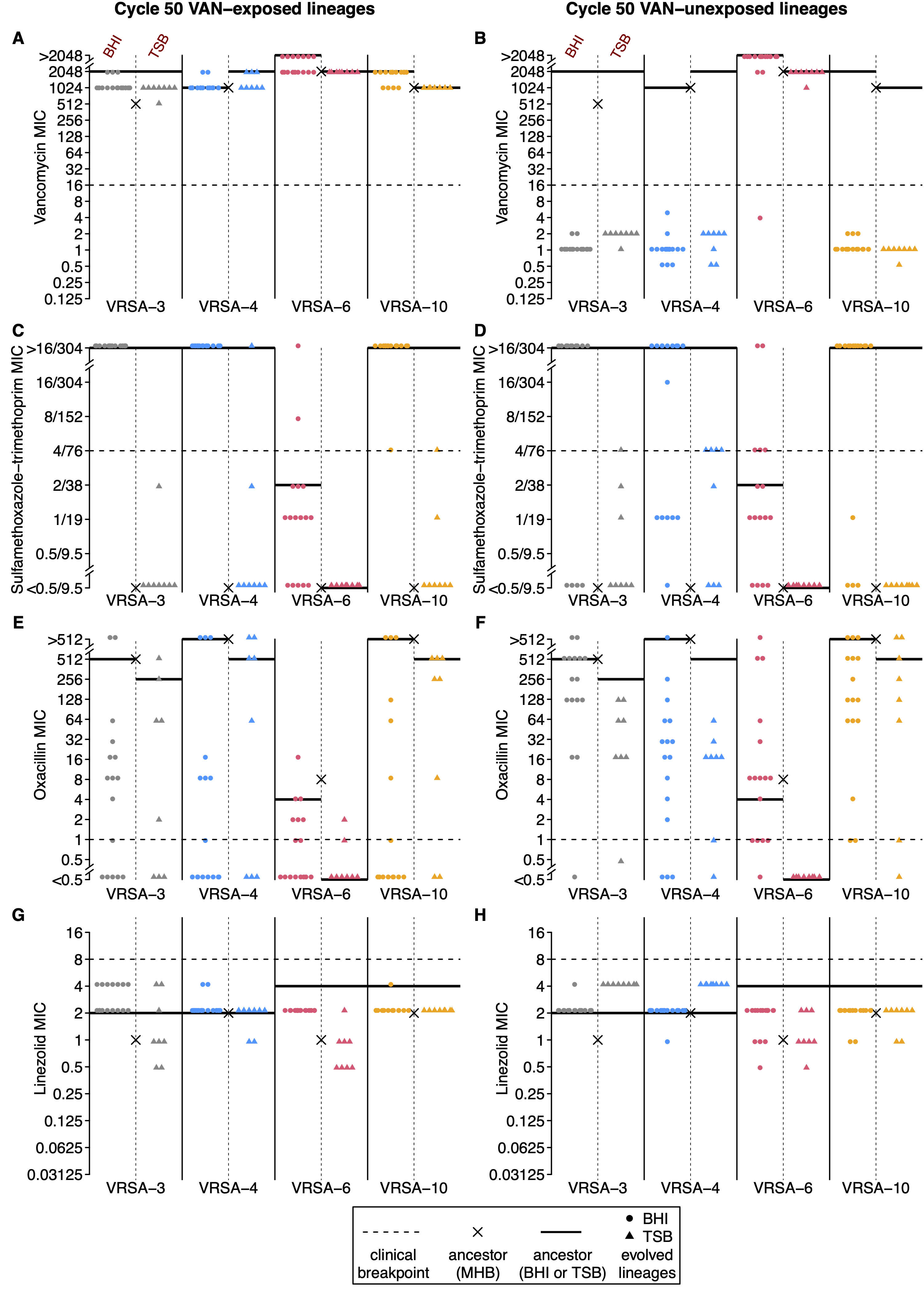

Supplement: S8 Fig — Horizontal dashed lines indicate the clinical breakpoint for each drug as defined in CLSI M100. Black horizontal lines indicate the MIC of ancestral strains as measured by broth microdilution in BHI and TSB. Black points (x) on the vertical dashed lines indicate the MIC of ancestral strains as measured by broth microdilution in cation-adjusted MHB. All MICs are given in units of μg/ml. (A) Vancomycin MIC of VAN-exposed lineages remained unchanged. (B) Vancomycin MIC decreased sharply in many VAN-unexposed lineages with the exception of most VRSA-6 lineages. (C-D) Most BHI-propagated lineages were resistant to sulfamethoxazole-trimethoprim (SXT), while TSB-propagated lineages were susceptible. Ancestral strains were SXT resistant as measured in BHI/TSB, but not MHB, with the exception of VRSA-6. Y-axis labels indicate the concentration of sulfamethoxazole and trimethoprim (e.g., 0.5/9 refers to 0.5 μg/ml sulfamethoxazole and 9 μg/ml trimethoprim). (E-F) Oxacillin MIC decreased in most lineages, but the magnitude of change was different between VAN-exposed and VAN-unexposed lineages. VAN-exposed lineages generally had lower oxacillin MICs than VAN-unexposed lineages. (G-H) Linezolid MIC remained largely unchanged in VAN-exposed and VAN-unexposed lineages. (TIFF) [file ppat.1012422.s008.tiff]

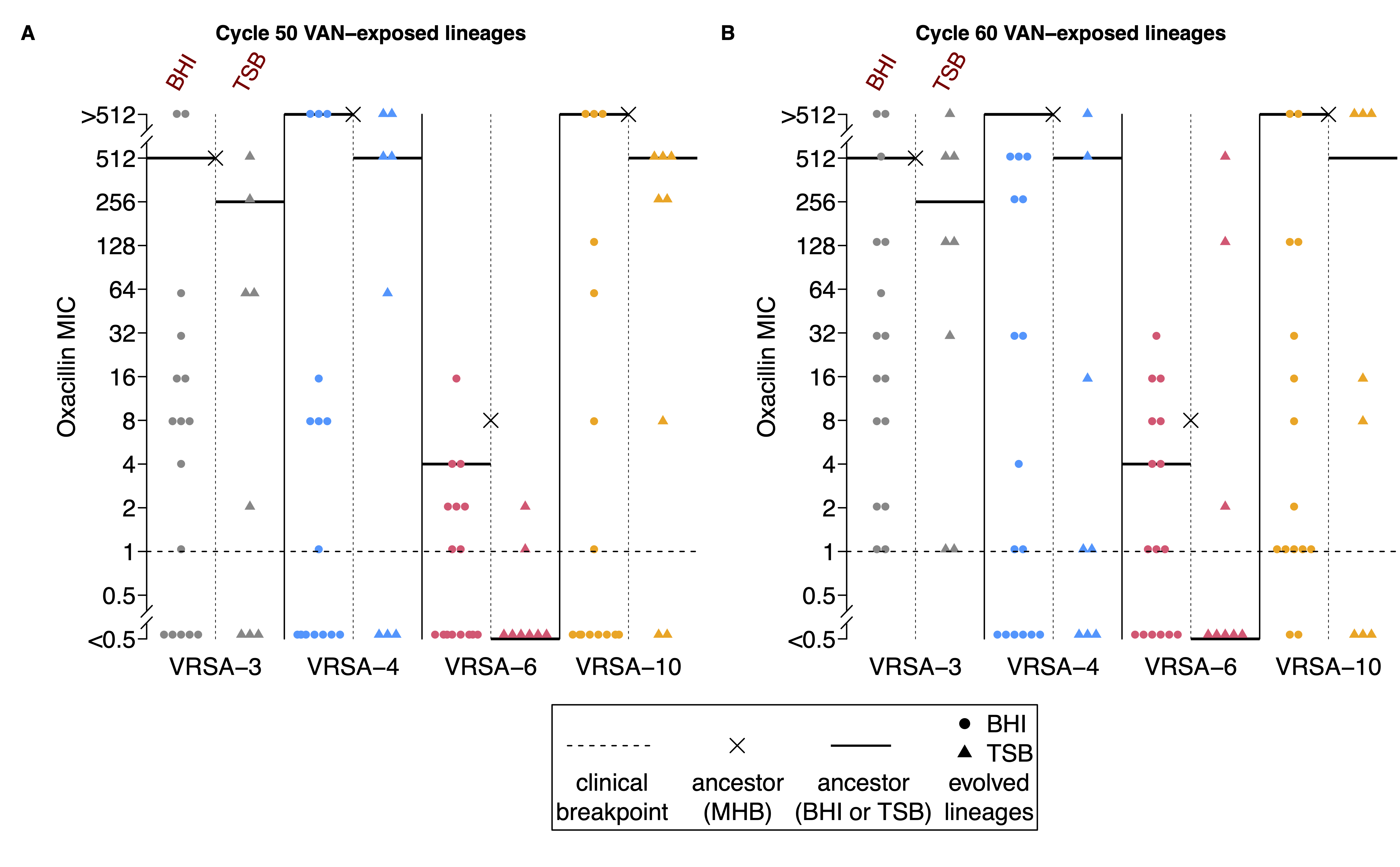

Supplement: S9 Fig — Horizontal dashed lines indicate the clinical breakpoint for each drug as defined in CLSI M100. Black horizontal lines indicate the MIC of ancestral strains as measured by broth microdilution in BHI and TSB. Black points (x) on the vertical dashed lines indicate the MIC of ancestral strains as measured by broth microdilution in cation-adjusted Mueller-Hinton Broth. All MICs are given in units of μg/ml. Oxacillin MICs increased slightly between (A) cycle 50 and (B) cycle 60 (two-sided Wilcoxon signed-rank test p-value = 0.023). (TIFF) [file ppat.1012422.s009.tiff]

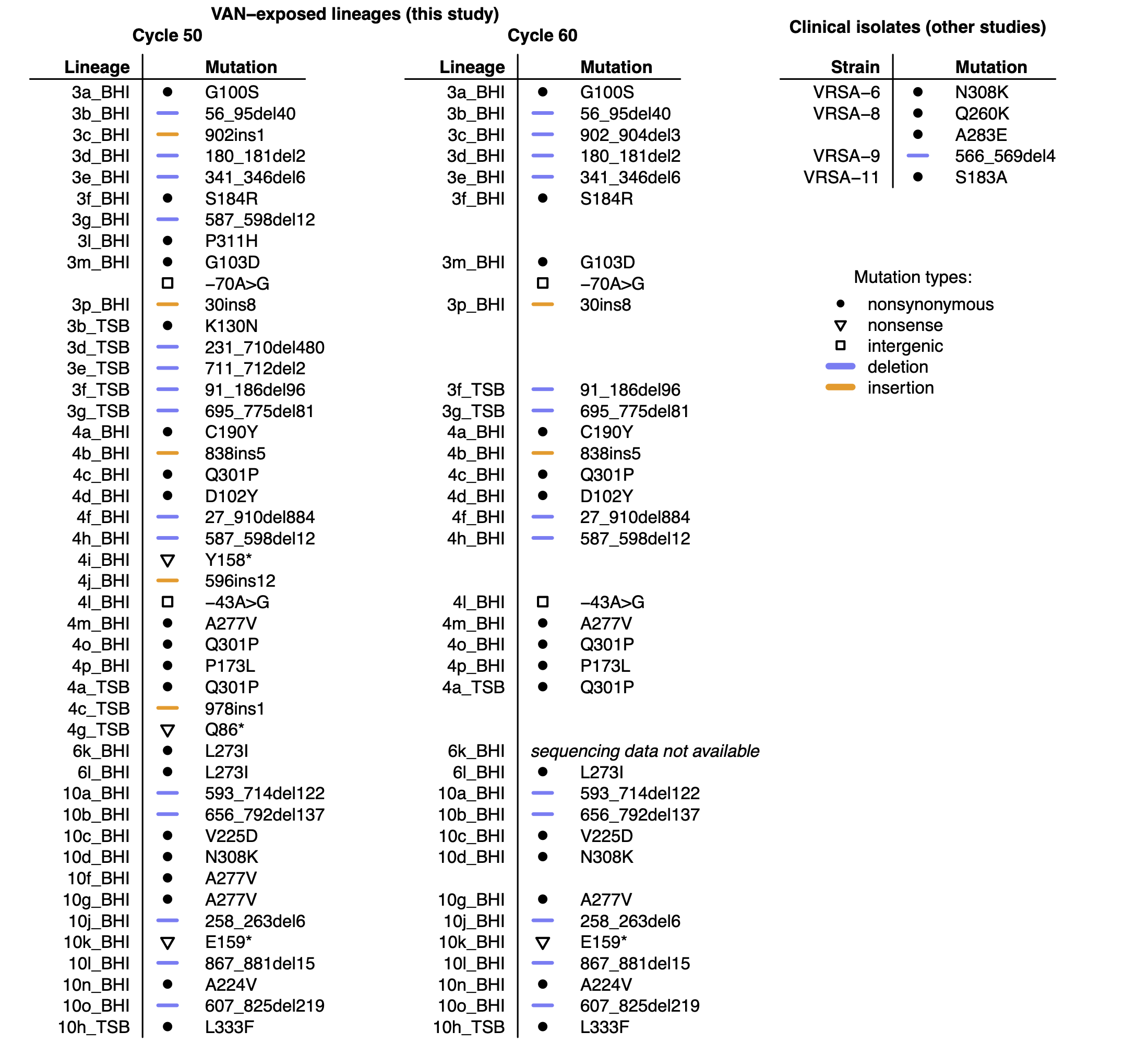

Supplement: S1 Table — Descriptions of the ddl mutations observed in VAN-exposed lineages generated in this study and in clinical VRSA isolates for which sequencing data is available (VRSA-1 through VRSA-11). Of the 44 lineages with a ddl mutation at cycle 50, 33 retained the mutation at cycle 60. Sequencing data was not available for cycle 60 VRSA-6k_BHI, which had a ddl mutation at cycle 50. Notably, VRSA-3c_BHI harbored a 4 base deletion (relative to cycle 50) that restored the frame of ddl and resulted in the deletion of Q301 and the substitution I302L relative to the wild type sequence. Additionally, VRSA-10d_BHI harbored the same nonsynonymous mutation as ancestral VRSA-6 (N308K). Nonsynonymous and nonsense mutations indicate the affected amino acid position, while insertion, deletion, and intergenic mutations indicate the affected nucleotide(s) position(s). Base 1 is the ‘A’ in start codon ‘ATG’ (‘587_598del2’ indicates that 12 nucleotides at positions 587 through 598 were deleted, ‘-70A>G’ indicates that the 70th nucleotide upstream of the start base was substituted, etc.). (TIFF) [file ppat.1012422.s010.tiff]
